# Office hours and caesarean section: systematic review and Meta-analysis

**DOI:** 10.1007/s43999-022-00002-6

**Published:** 2022-06-22

**Authors:** Ilir Hoxha, Arber Lama, Genta Bunjaku, Krenare Grezda, Riaz Agahi, Petrit Beqiri, David C. Goodman

**Affiliations:** 1 The Dartmouth Institute for Health Policy and Clinical Practice, Geisel School of Medicine at Dartmouth, Lebanon, NH 03756 USA; 2 Kolegji Heimerer, 10000 Prishtina, Kosovo; 3 Evidence Synthesis Group, 10000 Prishtina, Kosovo

**Keywords:** Caesarean section, Delivery time, Medical practice variation, Physician convenience, Practice patterns

## Abstract

**Background:**

Unnecessary caesarean births may be affected by physician factors, such as preferences, incentives and convenience. Delivery during office hours can be a valuable proxy for measuring such effects.

**Objective:**

To determine the effect of office hours on the decision for caesarean delivery by assessing the odds of caesarean during office hours compared to out-of-office hours.

**Search strategy:**

We searched CINAHL, ClinicalTrials.gov, The Cochrane Library, PubMed, Scopus and Web of Science from the beginning of records through August 2021.

**Data collection and analysis:**

Search results were screened by three researchers**.** First, we selected studies that reported odds ratios of caesareans, or data allowing their calculation, for office and out-of-office hours. We extracted data on the study population, study design, data sources, setting, type of caesarean section, statistical analysis, and outcome measures. For groups reporting the same outcome, we performed a standard inverse-variance random-effects meta-analysis, which enabled us to calculate the overall odds ratios for each group. For groups reporting varying outcomes, we performed descriptive analysis.

**Main results:**

Meta-analysis of weekday vs weekend for any caesarean section showed higher odds of caesarean during weekdays in adjusted analysis 1.40 (95%CI 1.13, 1.72 from 1,952,691 births). A similar effect was observed in the weekday vs Sunday comparison (1.39, 95%CI 1.10, 1.75, 150,932 births). A lower effect was observed for emergency CS, with a slight increase in adjusted analysis (1.06, 95%CI 0.90, 1.26, 2,622,772 births) and a slightly higher increase in unadjusted analysis (1.15, 95%CI 1.03, 1.29, 12,591,485 births). Similar trends were observed in subgroup analyses and descriptive synthesis of studies examining other office hours outcomes.

**Conclusions:**

Delivery during office hours is associated with higher odds for overall caesarean sections and little to no effect for emergency caesarean. Non-clinical factors associated with office hours may influence the decision to deliver by caesarean section. Further detailed investigation of the “office hours effect” in delivery care is necessary and could lead to improvements in care systems.

**Funding:**

The authors received no direct funding for this study.

**Supplementary Information:**

The online version contains supplementary material available at 10.1007/s43999-022-00002-6.

## Introduction

Since its introduction in medical practice, delivery by caesarean section (CS) has been associated with improved outcomes for mothers and infants [1]. While rates of 10-15% have been suggested as optimal, [2, 3] rates continue to surge in many countries beyond these widely accepted thresholds. In 2015, the global CS rate was estimated to be 21.1%,[4] with 2018 estimates of up to 17 million unnecessary CS [4]. Caesarean birth rates vary considerably between countries, with rates as low as 5% and as high as 58% [4, 5]. Like any other medical intervention, CS can have short and long term risks for mother and child [6]. Various studies have associated caesarean sections with an increased risk for stillbirth in subsequent childbirths, [7] placenta accreta spectrum, [8, 9] or diabetes development in childhood [10]. Moreover, overuse of caesarean sections is expensive, potentially wasting hospital resources and physician time [11–14].

The association of CS with health system factors has been investigated, and various factors appear to influence CS rates, including country-level factors [4, 12, 15, 16] such as cultural preferences, insurance systems, payment practices; [4, 17–19] hospital-level characteristics, such as for-profit or teaching status [17, 19–21] and micro-level factors, i.e. factors related to physician, [22] patient and clinical units [17, 23]. It has also been suggested that the delivery time may be associated with variation in CS rates [23, 24]. While this variation may result from the natural delivery cycle, [24–26] studies suggest health system factors may also be responsible [23, 24]. If we consider Wennberg types of care, CS, when clinically indicated, can be considered effective care, [27] important for the health of delivering mothers and their newborns. Nevertheless, it could also fall into the domain of preference and supply sensitive care when used without clear clinical indications.

In a hypothetical natural cycle of delivery, the CS rates should be the same within a given population or show only minor variation by time of day. However, in reality, higher CS rates are often observed during office hours (working days or hours). This effect may relate to physician and patient preferences or availability of maternity services, or a mixture of both. For example, CS overuse may reflect physician convenience, [23, 24, 28] i.e., it is easier to perform a CS during office hours. CS overuse during office hours may also reflect resource availability to perform operative work, or stronger incentives that are enacted via hospital strategies and arrangements, [28–30] and/or physicians’ incentives, [31] to perform more CS. Other factors may be relevant, including patient preferences, [32] manipulation of timing, [33] or physician practice style, which can be influenced by training and education [34]. We have included a conceptual framework for how office hours may influence CS rates (Additional file 1). Another critical hypothesis related to Wennberg types of care is that the office hours effect should be more prominent if clinical indications are not clear and less prominent if clinical indicators are unambiguous, i.e., when CS is the most effective or safest choice of care. For example, when there is an emergency during birth, i.e. foetal malposition or dystocia, there is little room for variation in clinical decision-making -- an emergency CS is often required. In cases where there are no medical indications for performing CS, some physicians may be more prone to perform one due to the influence of preference or supply factors, while others may be more bound to clinical criteria resulting in considerable variation in CS use. To explore this “office hours effect” and underlying factors at greater depths, we examined the relationship between time of delivery and CS through a systematic review of the relevant literature. This work sheds light on the effect of non-clinical factors on CS rates which could help address the overuse of CS in delivery care.

## Materials and methods

The study was modelled after our previous meta-analyses [18, 19, 21, 22, 35] and was also designed to adhere to existing guidelines: the Preferred Reporting Items for Systematic Reviews (PRISMA),[36, 37] Conducting Systematic Reviews and Meta-Analyses of Observational Studies of Aetiology (COSMOS-E) [38] and Meta-analysis Of Observational Studies in Epidemiology (MOOSE) guidelines [39]. Prior to study initiation, the protocol was submitted to Prospero (CRD42020158434).

### Search strategy and study inclusion criteria

We searched six databases: CINAHL, ClinicalTrials.gov, The Cochrane Library, PubMed, Scopus and Web of Science. We also augmented this with a manual search of the references of included studies. Our search strategy (available in the Additional file 1) was designed to obtain the broadest possible cross-section of data and was last updated in August 2021. Studies were excluded only to remove duplicate data. Exclusion criteria based on year of publication, country or language were not applied. We included studies that reported any, i.e. overall, or emergency CS by day of week or time of the day. The primary outcome was the odds ratio (OR) of CS by delivery time. Studies not reporting odd ratios but with information that allowed their calculation were also included. The suitability of studies for inclusion was assessed by independent reviewers (AL, GB, KG) by screening titles and abstracts followed by a full-text review. Any disagreements between reviewers were resolved by discussion or consultation with a senior reviewer (IH).

### Data extraction

Data extraction from included studies was performed independently by three researchers (AL, GB and KG) and reviewed by a senior researcher (IH). A data extraction spreadsheet was designed to include the independent variable information, OR and confidence limits, sample size, covariates used for statistical adjustment, data on the study population and other characteristics of the included studies. We assessed all studies for risk of bias across six domains according to the Quality in Prognosis Studies (QUIPS) protocol [40]. Each study was assigned a risk of bias (low, medium or high) within six domains – study participation, study attrition, prognostic factor measurement, outcome measurement, study confounding, and statistical analysis and reporting.

### Data analysis

The primary outcomes of interest were odds ratios and rates of CS. We report separate effect estimates for “any” or “emergency” CS. This distinction is critical as “any” CS rate includes planned CS, likely to be performed during office hours [41]. If CS occurred during labour, it would be considered an emergency CS, whereas if CS occurred without labour, it would be considered a planned CS. Using these types of CS outcomes was necessary to examine our study hypothesis. Any CS would show office hours effects likely caused by preference and supply factors. On the other hand, emergency CS, because it should be based solely on medical indications, should not be associated with office hours effects. However, observed effects may reflect practice patterns in dealing with medical indications for CS.

The primary variable of interest was time of delivery (office hours compared to out-of-office hours) as a proxy variable indicating preference and supply effects. The included studies examined the effect of office hours on CS using a variety of comparisons for the time of the day. For this reason, we assigned the effect estimates reported by studies to different groups: day vs night, evening vs night, day and evening vs night, day vs evening, weekday vs weekend, weekday vs Sunday, and office hours vs out-of-office hours, where ‘office hours’ denotes working hours during weekdays. The time classification of day, evening or night was not identical among the studies. As a result, we performed two types of analysis. First, we have performed a meta-analysis for studies where comparison groups matched (i.e. weekday vs weekend and weekday vs Sunday). We then performed a descriptive analysis without estimating an overall effect for other groups that did not report comparable delivery time comparisons. The analysis (meta-analysis or descriptive analysis) was performed for each comparison group separately.

We performed the meta-analysis for adjusted and crude estimates. Standard inverse-variance random-effects meta-analysis was used to obtain an overall OR. An overall OR lower than 1 denotes a decreased likelihood of CS during office hours. The heterogeneity of studies was determined using τ^2^. Values of 0.04, 0.16 and 0.36 represent low, moderate and high heterogeneity between studies [42]. For adjusted estimates, we conducted subgroup analysis by country, study design, CS rate, period of data collection, population by Robson groups and criteria, inclusion in the sample of women with existing conditions, type of data used and the domains of (QUIPS) risk of bias. Robson identifies ten groups using the onset of labour, presentation of foetus, previous CS, number of neonates and gestational age as criteria for classification [43]. We chose Robson groups and criteria because it is a well-known and easy to interpret metric valuable for identifying the risk for birth outcomes and the overuse of CS. In analysis, we use Robson groups and criteria because many studies do not report data by Robson groups, or report them for multiple groups. Having additional analysis by Robson criteria can somewhat compensate for this. In addition, as a measure of potential risk for CS, we have used an additional variable of whether women with existing conditions were included in the study sample.

Descriptive analysis was carried out for comparisons of: day vs night, evening vs night, day and evening vs night, day vs evening and office hours vs out-of-office hours. This included a presentation of effect estimates in a forest plot without meta-analysis. Together with effect estimates for each study, we present information on the country, sample size, study design, period of data collection, CS rate (if available), population by Robson group, source population, the inclusion of women with existing conditions in the study sample, and inclusion of births with conditions developed during delivery. The source population variable distinguishes if the sample was a sample of any risk cases or high-risk cases such as dystocia, gestational diabetes (GDM) and existing diabetes (GDM*) or second stage of labour malposition. The other two variables reported if the study sample included or excluded the women with existing conditions or conditions developed during pregnancy, both useful indicators for the inclusion of a higher risk population in the study sample.

Finally, we have graphically represented CS rates for office hours and out-of-office hours for outcomes (Any or Emergency CS) and comparisons (office hour categories).

Statistical analysis was carried out using STATA, release V.17 BE (StataCorp).

## Results

**Fig. 1:**
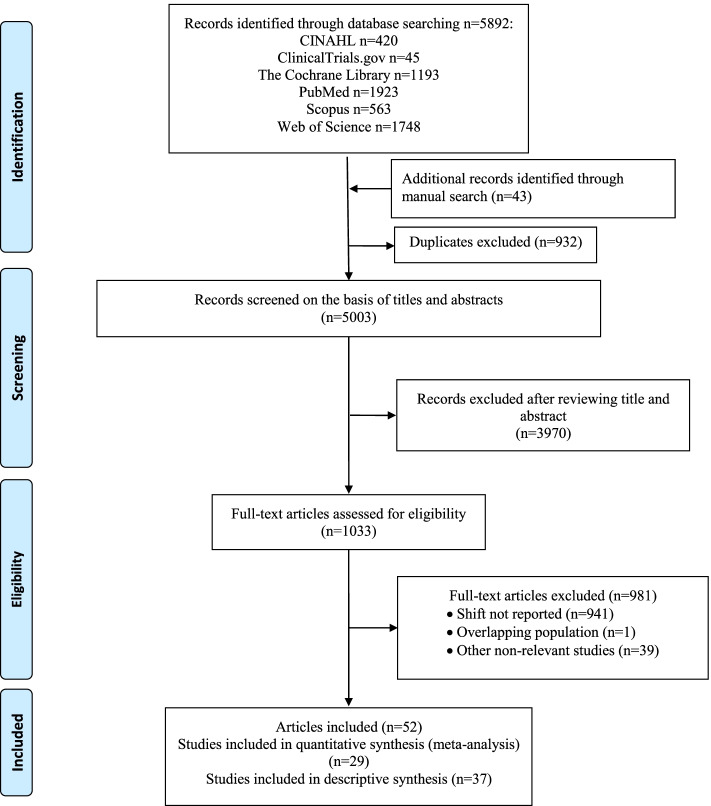
The flow diagram of review

We identified 5935 records; 420 from CINAHL, 45 from ClinicalTrials.gov, 1193 from The Cochrane Library, 1923 from PubMed, 563 from Scopus, 1748 from Web of Science; and 43 from manual search (Fig. 1). We removed 932 duplicates. The remaining 5003 records were screened for eligibility by review of titles and abstracts, of which 3970 were excluded based on their focus on other medications or surgeries. The remaining 1033 articles were screened in full text. We removed studies which did not report shift (941), overlapping populations (1) and other irrelevant studies (39). We were finally left with 52 articles (53 studies), from which 29 studies were suitable for quantitative synthesis (meta-analysis) and 37 studies were suitable to be included in descriptive analysis [23, 24, 28, 31, 33, 34, 41, 44–89].

Characteristics of included studies are presented in Additional file 1. Studies were analysed with a focus on the circumstances surrounding delivery, type of CS, and the types of pregnancy according to the Robson Classification [90]. These studies were carried out in a diverse range of countries, with the population size of studies ranging from 266 to 8.62 million births, during the years 1982 to 2019 with most of them using hospital data. Study populations and variables used for statistical adjustment varied considerably.

### Risk of bias of included studies

Confounding was the main source of bias for studies that reported any CS. Eight out of 32 studies had a moderate risk of bias, while 17 studies had a high risk of bias from confounding. Apart from a moderate risk of bias concerning study participation for three studies, all other studies had a low risk of bias across all remaining QUIPS domains. Similarly, studies across all domains had a low risk of bias except for confounding for emergency CS. In this domain, out of 22 reporting emergency CS, one study had a moderate risk of bias, and 15 had a high risk of bias.

### Any caesarean section

Any CS was examined using 32 studies reported in 31 articles.

#### Meta-analysis

**Fig. 2:**
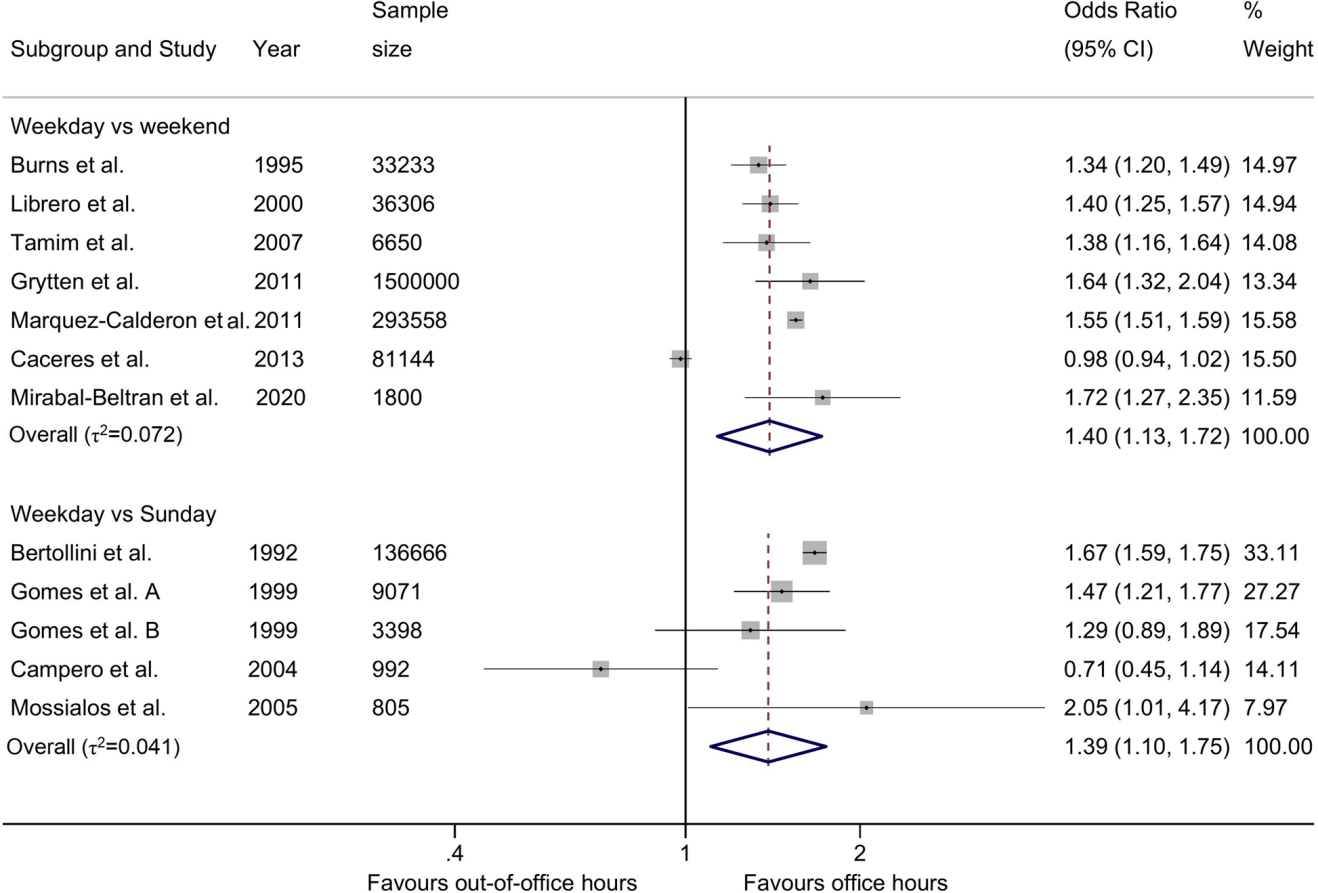
Adjusted odds ratios of any caesarean section

Figure 2 presents meta-analyses for the adjusted ORs of any CS, comparing weekdays with weekends (7 studies, 1,952,691 births) and weekdays with Sundays (5 studies, 150,932). We found that the odds of CS were 1.40 (95%CI 1.13, 1.72) during weekdays as compared to weekends and 1.39 (95%CI 1.10, 1.75) during weekdays as compared to Sundays, with low heterogeneity between studies (τ2 = 0.072, τ2 = 0.041).

**Fig. 3:**
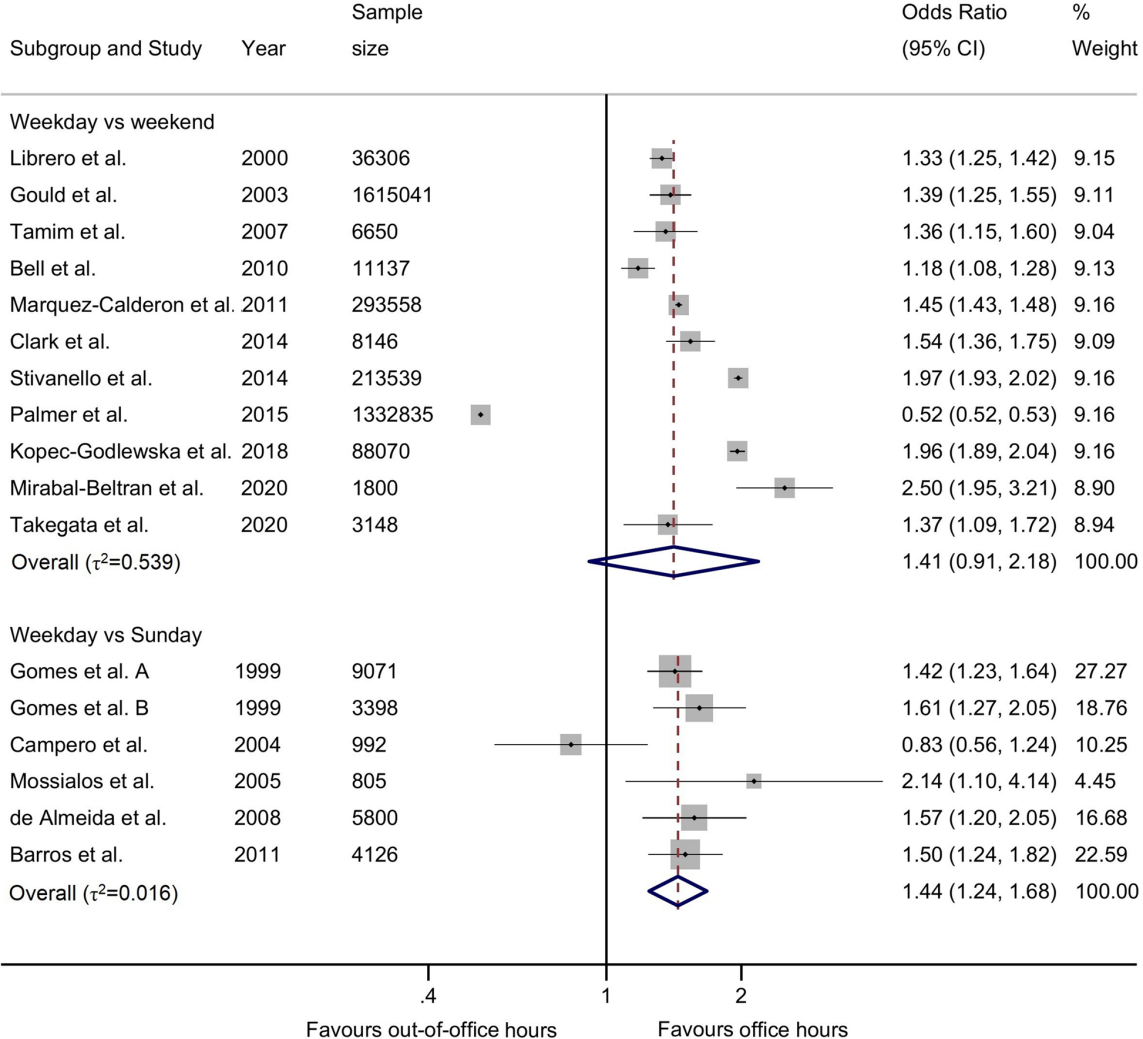
Crude odds ratios of any caesarean section

Figure 3 displays the unadjusted analysis for weekday vs weekend analysis. Across 11 studies (3,610,230 births), we observed a higher OR for weekdays (1.41 95%CI 0.91, 2.18), with high heterogeneity among studies (τ2 = 0.539). Notably, one study by Palmer et al. showed lower odds of any caesarean during weekdays (0.52, 95%CI 0.52, 0.53), while all other studies reported higher odds of CS on weekdays. In the unadjusted analysis of 6 studies (24,192 births), comparing weekdays with Sundays, we found higher odds of any caesarean section on weekdays (1.44, 95%CI 1.24, 1.68) with no heterogeneity between studies (τ2 = 0.016).

#### Subgroup analysis of adjusted effect estimates

For weekdays and weekends comparison (Additional file 1), the higher odds during weekdays were present in all subgroups, including groups indicating clinical risk or other study characteristics. Tests for interaction across all subgroups were negative. Subgroup effect estimates ranged from 1.15 to 1.72. For example, the OR of CS in Robson groups 1 to 5 was 1.27 (95%CI 0.73, 2.21), for studies reporting data for all Robson groups, the OR was 1.47 (95%CI 1.36, 1.60), and for studies reporting data for multiple Robson groups, the OR was 1.38 (95%CI 1.16, 1.64). Studies with CS rates below or equal to 19% had an OR of 1.40 (95%CI 1.25, 1.57), those with rates of 20-40% the OR was 1.51 (95%CI 1.38, 1.66), and studies with CS rates over 40% the OR was 1.72 (95%CI 1.27, 2.35). In subgroup analysis for weekdays vs Sundays comparison, we also observed higher odds for CS, except for Mexico, studies reporting period before and after 2000, and studies not reporting CS rates. Subgroup effect estimates ranged from 0.71 to 2.05. Tests for interaction were positive for country (p for interaction = 0.001), period of data collection (p for interaction = 0.004) and CS rate (p for interaction = 0.003) (Additional file 1).

#### Descriptive analysis

Studies showed higher odds of CS during the days as compared to nights in all studies reporting adjusted estimates, with values ranging from 1.07 (95%CI 1.03, 1.12) [53] to 3.45 (95%CI 1.95, 6.09) [31] (Additional file 1). Studies also showed higher odds of CS during the evenings than during night shifts. Effect sizes varied from 1.40 (95%CI 0.48, 4.06) [59] to 4.65 (95%CI 3.13, 6.92) [65]. Single studies for day vs evening comparison (1.35, 95%CI 1.22, 1.49) [34] and office vs out-of-office hours comparison (2.36, 95%CI 1.37, 4.06), [23] again showed higher odds in daytime and office hours respectively. In the crude estimates, we observed similar trends. All included studies in a day vs night, evening vs night, day and evening vs night, and office vs out-of-office hours comparisons reported higher odds of CS during the day and/or evening, and office hours. Only one study reported a lower OR in the day vs evening comparison (Additional file 1) [80].

### Emergency caesarean

Emergency CS was examined using 22 studies reported in the same number of articles.

#### Meta-analysis

**Fig. 4:**
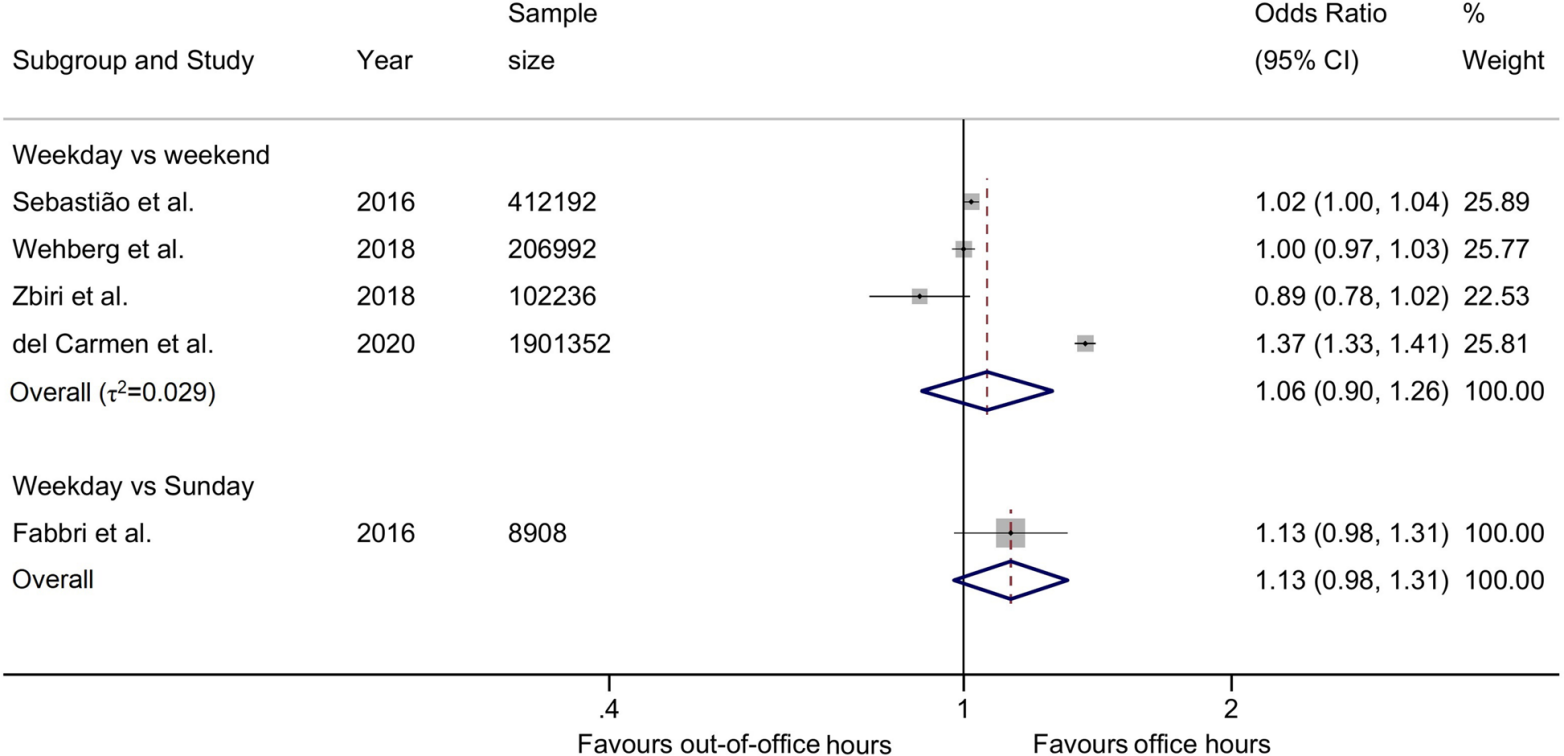
Adjusted odds ratios of emergency caesarean section

**Fig. 5:**
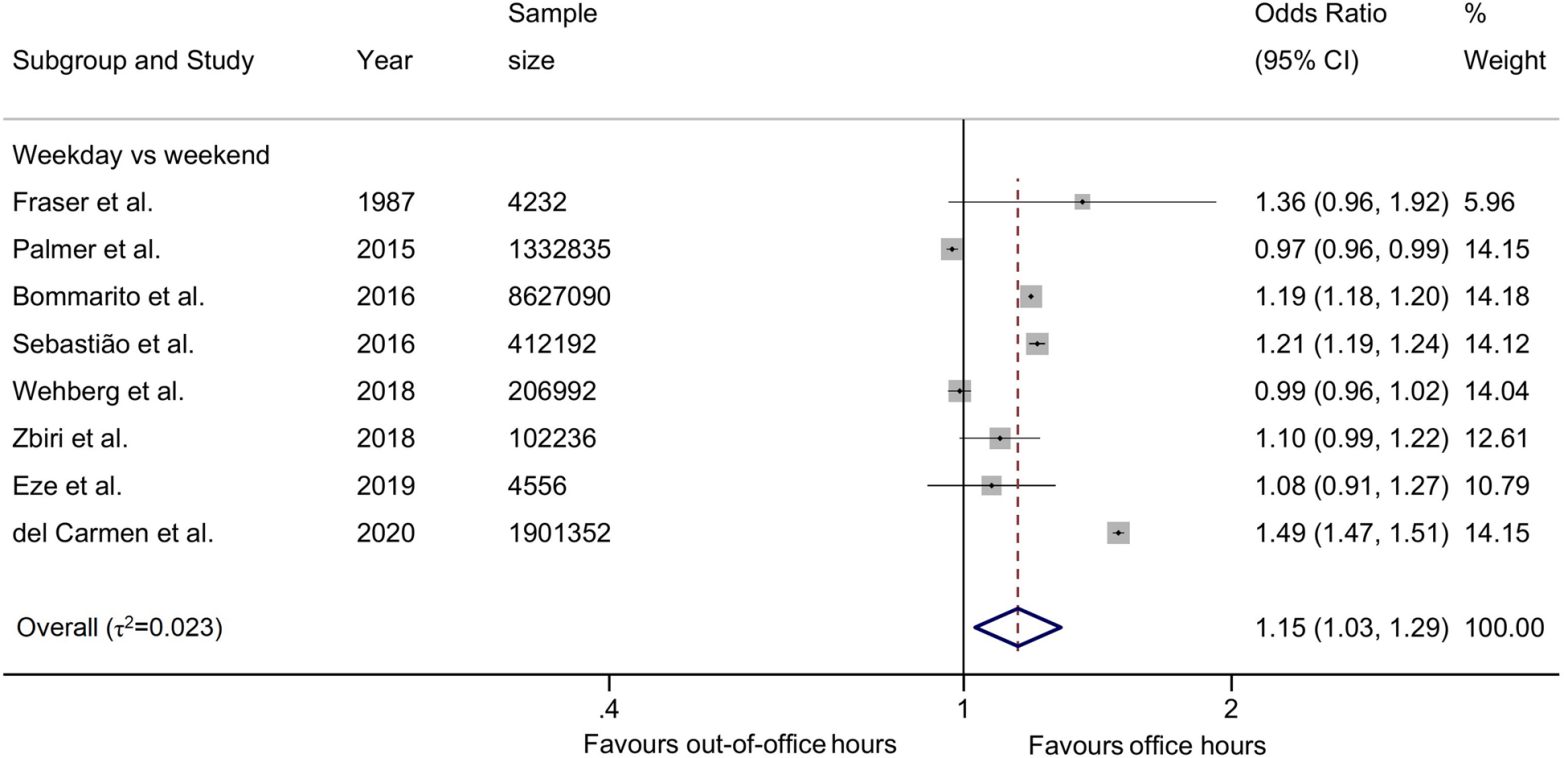
Crude odds ratios of emergency caesarean section

There was a small difference in the odds of emergency CS on weekdays vs weekends comparison across four studies (2,622,772 births), with an overall odds ratio of 1.06 (95%CI 0.90, 1.26) and no relevant heterogeneity between studies (τ2 = 0.029, Fig. 4). The odds ratios varied between 0.89 (95%CI 0.78, 1.02) [86] to 1.37 (95%CI 1.33, 1.41) [58]. Only one study (8908 births) reported weekdays vs Sundays and showed higher odds during weekdays at 1.13 (95%CI 0.98, 1.31) [33]. In the analysis of unadjusted estimates from 8 studies (12,591,485 births), we found higher odds of CS on weekdays as compared to weekends (1.15, 95%CI 1.03, 1.29) with no relevant heterogeneity between studies (τ2 = 0.023, Fig. 5).

#### Subgroup analysis

There was considerable variation between pooled effect estimates among different subgroups for weekday vs weekend comparison. There was a positive test for interaction for the subgroups of women with existing conditions, birth registry data, and claims data. Pooled effect estimates ranged from 0.89 (95%CI 0.78, 1.02) to 1.37 (95%CI 1.33, 1.41). For example, in studies excluding women with existing conditions, the OR of CS was 1.37 (95%CI 1.33, 1.41), while the odds reported in studies including women with existing conditions the OR was 1.00 (0.97, 1.04). For studies with CS rates below or equal to 19% the OR was 1.17 (95%CI 0.86, 1.59) and with studies reporting rates of 20-40% the OR was 0.97 (95%CI 0.85, 1.10, Additional file 1).

#### Descriptive analysis

Adjusted ORs for day vs night comparison varied considerably in effect sizes. A study reported lower rates of emergency CS during the day (0.67, 95%CI 0.60, 0.75) [55]. Higher odds during daytime were reported by Sebastião and co-workers (1.19, 95%CI 1.16, 1.22) [81]. Higher odds were also reported for evenings compared to nights (1.50 95%CI 1.46, 1.54) [81] and office compared to out-of-office hours (1.06, 95%CI 1.02, 1.11) [23]. Trends were similar for unadjusted estimates.

### CS rates analysis

#### Any caesarean

All studies showed higher CS rates during daytime compared to night. In addition, higher rates of CS during the day or weekday were observed for almost all studies in the day vs evening, weekday vs weekend comparison and all the studies in weekdays vs Sunday comparisons (Additional file 1). The CS rate was also considerably higher for office hours in a single study comparing office with out-of-office hours.

#### Emergency caesarean

We found a negligible difference between office and out-of-office hours comparisons for emergency CS rates. CS rates were often very close to each other and even lower during office hours.

## Discussion

All meta-analyses reveal that women are more likely to undergo a caesarean delivery during office hours for any CS outcome, which is consistent with individual studies included in the descriptive analysis. The results of our meta-analysis for emergency CS show a small effect in adjusted, crude and subgroup analysis. Descriptive analysis of individual papers in other group comparisons shows variation in effect estimates below and above value 1.

### Strengths and limitations

The major strengths of our meta-analysis include a broad literature search, screening and data extraction performed by multiple reviewers, an exploration of study characteristics as a potential source of variation between studies, and quality assessment using the QUIPS tool. We were able to include many studies with a large number of births. Most of the studies included in the meta-analysis used data from after 2000. Having more recent data is more beneficial for current discussions on CS rates. To consider the effect of medical risk for CS, we classified pregnancies according to Robson criteria and other clinical risk criteria, which were then used in subgroup analysis. The use of such clinical criteria enabled the assessment of the risk of pregnancy for study population with unambiguous and widely accepted categories. The challenge with Robson criteria was that most of the study populations belonged to multiple Robson groups, making it difficult to utilise fully such classification in subgroup analysis. The potential risk of bias in our review lies in the use of effect estimates that lacked control for known confounders. Many studies included in the review reported a moderate or high risk of bias for the confounding domain. The included papers also varied in terms of study designs. Using studies with different study designs can lead to biases associated with design features such as participant selection, sampling and data measurement. For this reason, we also examined and reported the study design in subgroup analysis. Variation in the study definitions of office hours was another major challenge which was solved with the organization of studies in different subgroups and performing only a descriptive analysis for studies that reported varying times.

### Interpretation of the findings

Our analysis shows that regardless of clinical risk and other factors we examined, the odds for any CS were higher during office hours. This apparent CS overuse during office hours may be explained by preference and supply influences [23, 24, 28–31, 33, 59, 60, 62, 85, 91]. “Office hours effect” is likely a manifestation of many factors such as application of clinical guidelines, medical practice style, and induction to speed up the delivery process, as examples of preference-related factors. Financial incentives, availability of resources and other support are examples of supply-related factors that can contribute to such “effect” as well. Even factors such as patient behaviour, preferences [23, 46, 92] or convenience [24, 93–95] and strategies of clinicians to reduce complications and to avoid litigation can intermingle in the process [64, 96–101]. In contrast, the analysis shows negligible effect sizes for emergency CS indicating that when CS is the effective care option, the decision for CS is little affected by office hours. The variation in effect estimates for emergency CS during office hours in different studies may be an effect of inappropriate care, practice patterns among clinicians or medical reasons [23, 24, 28, 29, 31]. As a result, in any interpretation of the “time effect” on CS, we must consider the proxy nature of office hours as a variable which represents many of the above-highlighted effects.

### Implications for health policy and clinical practice

Close to a third of primary caesarean deliveries in low-risk women could be avoided by altering the current maternity care practices [24, 98, 99]. The results of this study could help in the first step of avoiding unnecessary CS and improvement of systems of maternity care, that is, in understanding of how health system factors influence delivery care. More specifically, this study contributes to understanding of the role of preference and supply factors related to the “office hours effect” in the CS decisions. This review may also encourage review of clinical practice differences during office and out-of-office hours among clinicians, hospitals, regions and countries. The examination of the underlying causes behind the “office hours effect” will help identify real causes of this CS variation and direct remedies for addressing it. For example, physician preferences may be remedied with a consistent application of clinical guidelines and review of clinical decision making. Overuse due to financial incentives can be addressed with a review and change of payment mechanisms.

### Implications for research

This review also provides information for the design of future studies and reviews of clinical decision making related to office hours effect on CS. In quantitative studies, we recommend that the effect of office hours be examined by level of patient risk, such as Robson groups or criteria, women with pre-existing conditions and conditions developed during pregnancy, i.e. material and/or foetal complications. This approach will limit confounding and provide greater clinical meaning to the analytical outcomes. Qualitative methods could also be useful in the examination of the “office hours effect”. Qualitative studies at the country, regional, or hospital level could help to distinguish the effects of specific underlying factors, only hinted in the existing research.

## Conclusion

Delivery during office hours is associated with higher odds for caesarean sections overall and, to a lesser extent, for emergency caesarean. This review can enhance our understanding of non-clinical factors that influence caesarean rates, in particular the timing of caesareans. This review should encourage review and improvements in delivery care and support research that examines the underlying causes and outcomes of the “office hours effect” on caesarean section rates.

### Supplementary Information

**Additional file 1.**

## Data Availability

All authors have completed the ICMJE uniform disclosure form at www.icmje.org/coi_disclosure.pdf and declare: no support from any organization for the submitted work; no financial relationships with any organizations that might have an interest in the submitted work in the previous 3 years; no other relationships or activities that could appear to have influenced the submitted work. All authors had full access to all of the data (including statistical reports and tables) in the study and take responsibility for the integrity of the data and the accuracy of the data analysis.
