# Supplementary material for: Office hours and caesarean section: systematic review and Meta-analysis
Source: Res Health Serv Reg. 2022 Jun 22;1:4. doi: 10.1007/s43999-022-00002-6 (PMC11264882; doi:10.1007/s43999-022-00002-6)
Supplement: Supplementary file 1 — Additional file 1. [file 43999_2022_2_MOESM1_ESM.pdf]

A conceptual framework for influence of office hours in CS rates

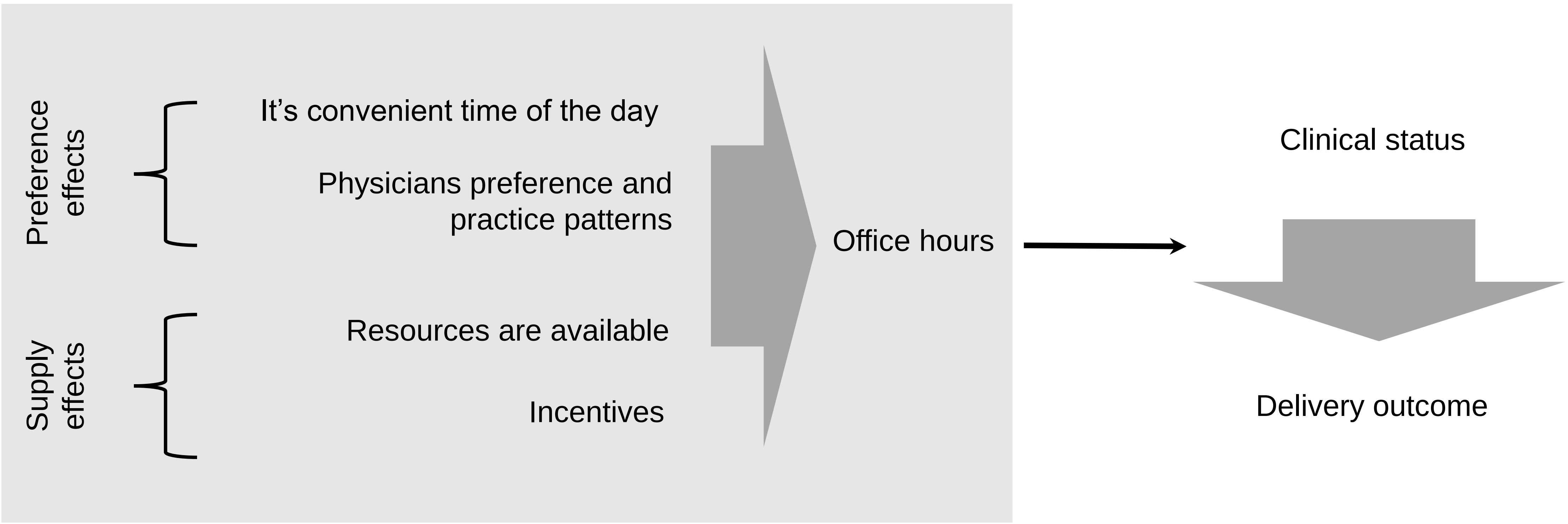

For Medline (PubMed)
https://www.ncbi.nlm.nih.gov/pubmed/
(((((((causes OR determinants OR factors OR decision* OR physician* OR midwife* OR gender OR socioeconomic OR evidence-based OR hospital OR hospitals OR teaching hospital OR uncertain* OR educational status OR social class OR race OR convenience OR shift OR obstetric* OR gynecolog* OR health system OR supply OR distribut* OR utilization OR practice OR insurance OR choice OR attitude OR patient OR maternal OR accessib* OR health service* [Title/Abstract])) NOT medline[sb])) OR ("Decision Making"[Mesh] OR "Physician's Practice Patterns"[Mesh] OR "Socioeconomic Factors"[Mesh] OR "Evidence-Based Medicine"[Mesh] OR "Hospitals"[Mesh] OR "Uncertainty"[Mesh] OR "Educational Status"[Mesh] OR "Hospital Costs"[Mesh] OR "Physician Incentive Plans"[Mesh] OR "Social Class"[Mesh] OR "Obstetrics and Gynecology Department, Hospital"[Mesh] OR "supply and distribution"[Subheading] OR "utilization"[Subheading] OR "Insurance"[Mesh] OR "Choice Behavior"[Mesh] OR "Attitude to Health"[Mesh] OR "Patient Participation"[Mesh] OR "Physician-Patient Relations"[Mesh] OR "Economics, Hospital"[Mesh] OR "Maternal Health Services"[Mesh] OR "Health Services Accessibility"[Mesh]))) OR factors OR causes OR determinants AND (((((operative delivery OR caesarean section OR cesarean section OR c-section OR c section OR caesarean OR cesarean OR caesarean delivery OR cesarean delivery)))) OR cesarean section [MeSH Terms])) AND (((("Health Services Research"[Mesh]))) OR ((((medical practice variation OR regional variation OR regional rates OR hospital variation OR hospital rates))))) AND (“Cohort Study” OR “Studies, Cohort”, OR “Study, Cohort” OR “Cross Sectional Studies” OR “Cross-Sectional Study” OR “Studies, Cross-Sectional” OR “Study, Cross-Sectional” OR review OR “systematic review”)

General data						Characteristics of included studies						
Author	Year	Determinant being compared	Determinant details (hour)	Reference	Reference (hour)	Country	Study design	Number of cases	Number of hospital units	Year of data collection	Sampling	Type of CS analysed
**Any CS**												
**Day vs night**												
Hall et al.	1982	Day	06:01 to 18:00	Night	00:00 to 06:00	United States	Prospective	878	1	1980	Consecutive	Any
Busowski et al.	1997	Day	07:01 to 17:00	Night	00:00 to 07:00	United States	Retrospective	3449	1	1990-1991	Consecutive	Any
Gomes et al. B	1999	Day	07:00 to 18:00	Night	01:00 to 06:00	Brazil	Prospective	2846	10	1994	Consecutive	Any
Mitler et al.	2000	Night	n/a	Day	n/a	United States	Retrospective	1000	1	1990-1995	Random	Any
Lee et al.	2003	Day	08:00 to 17:00	Night	17:00 to 08:00	Canada	Retrospective	5192	17	1996-1997	Consecutive	Any
Campero et al.	2004	Day	08:00 to 15:59	Night	00:00 to 07:59	Mexico	Retrospective	669	Not reported	1987-2000	Consecutive	Any
Gould et al.	2005	Night	19.00 to 06:00	Day	07:00 to 18:00	United States	Retrospective	3362890	Not reported	1992-1997	Consecutive	Any
Mossialos et al.	2005	Day	08:00 to 16:00	Night	00:00 to 08:00	Greece	Retrospective	612	3	2002	Consecutive	Any
D Orsi et al.	2006	Day	09:00 to 14:59	Night	00:00 to 05:59	Brazil	Retrospective	231	1	1998-1999	Random	Any
de Almeida et al.	2008	Day	06:00 to 17:59	Night	00:00 to 05:59	Brazil	Retrospective	4005	2	1999	Consecutive	Any
Bell et al.	2010	Night	00:00 to 07:59	Day	08:00 to 17:00	United States	Prospective	7559	17	2001-2005	Consecutive	Any
Osava et al.	2011	Night	00:00 to 06:59	Day	07:00 to 18:59	Brazil	Retrospective	1949	1	2005	Consecutive	Any
Caceres et al.	2013	Day	08:00 to 19:59	Night	20:00 to 07:59	United States	Retrospective	54299	49	2004-2006	Consecutive	Any
Stivanello et al.	2014	Day	07:01 to 18:59	Night	19:00 to 07:00	Italy	Retrospective	213539	36	2005-2010	Consecutive	Any
Brookfield et al.	2016	Night	00:00 to 06:59	Day	07:00 to 16:59	United States	Prospective	2928	13	2000-2001	Consecutive	Any
Frank-Wolf et al.	2016	Day	07:00 to 15:00	Night	23:00 to 07:00	Israel	Retrospective	37996	1	2005-2014	Consecutive	Any
Saraiva et al.	2017	Night	00:00 to 06:59	Day	07:00 to 18:59	Brazil	Prospective	301	1	2013	Consecutive	Any
Mirabal-Beltran et al.	2020	Night	18:00 to 05:59	Day	06:00 to 17:59	United States	Retrospective	1800	1	2010-2016	Consecutive	Any
**Evening vs night**												
Hall et al.	1982	Evening	18:01 to 24:00	Night	00:00 to 06:00	United States	Prospective	467	1	1980	Consecutive	Any
Burns et al.	1995	Night	00:00 to18:00	Evening	18:00 to 24:00	United States	Retrospective	33233	36	1989	Consecutive	Any
Busowski et al.	1997	Evening	17:01 to 23:59	Night	00:00 to 07:00	United States	Retrospective	3478	1	1990-1991	Consecutive	Any
Gomes et al. B	1999	Evening	19:00 to 24:00	Night	01:00 to 06:00	Brazil	Prospective	2846	10	1994	Consecutive	Any
Campero et al.	2004	Evening	16:00 to 23:59	Night	00:00 to 07:59	Mexico	Retrospective	622	Not reported	1987-2000	Consecutive	Any
Mossialos et al.	2005	Evening	16:00 to 24:00	Night	00:00 to 08:00	Greece	Retrospective	319	3	2002	Consecutive	Any
D Orsi et al.	2006	Evening	15:00 to 23:59	Night	00:00 to 05:59	Brazil	Retrospective	266	1	1998-1999	Random	Any
de Almeida et al.	2008	Evening	18:00 to 23:59	Night	00:00 to 05:59	Brazil	Retrospective	2792	2	1999	Consecutive	Any
Frank-Wolf et al.	2016	Evening	15:00 to 23:00	Night	23:00 to 07:00	Israel	Retrospective	36828	1	2005-2014	Consecutive	Any
**Day and evening vs night**												
Freitas et al.	2016	Day and evening	06:00 to 23:59	Night	00:00 to 05:59	Brazil	Retrospective	60904	61	2012	Consecutive	Any
**Day vs evening**												
Burns et al.	1995	Day	06:00 to 18:00	Evening	18:00 to 24:00	United States	Retrospective	33233	36	1989	Consecutive	Any
Bell et al.	2010	Evening	17:01 to 23:59	Day	08:00 to 17:00	United States	Prospective	8009	17	2001-2005	Consecutive	Any
Osava et al.	2011	Evening	19:00 to 23:59	Day	07:00 to 18:59	Brazil	Retrospective	1671	1	2005	Consecutive	Any
Brookfield et al.	2016	Evening	17:00 to 23:59	Day	07:00 to 16:59	United States	Prospective	3034	13	2000-2001	Consecutive	Any
Saraiva et al.	2017	Evening	19:00 to 23:59	Day	07:00 to 18:59	Brazil	Prospective	266	1	2013	Consecutive	Any
**Weekday vs weekend**												
Burns et al.	1995	Weekend		Weekday		United States	Retrospective	33233	36	1989	Consecutive	Any
Librero et al.	2000	Weekday		Weekend		Spain	Retrospective	36306	11	1994-1995	Consecutive	Any
Gould et al.	2003	Weekday		Weekend		United States	Retrospective	1615041	Not reported	1995-1997	Consecutive	Any
Tamim et al.	2007	Weekday		Weekend		Lebanon	Retrospective	6650	9	2001-2002	Consecutive	Any
Bell et al.	2010	Weekend		Weekday		United States	Prospective	11137	17	2001-2005	Consecutive	Any
Grytten et al.	2011	Weekend		Weekday		Norway	Retrospective	1500000	44	1967-2005	Consecutive	Any
Marquez-Calderon et	2011	Weekday		Weekend		Spain	Retrospective	293558	Not reported	2007-2009	Consecutive	Any
Caceres et al.	2013	Weekend		Weekday		United States	Retrospective	81144	49	2004-2006	Consecutive	Any
Clark et al.	2014	Weekday		Weekend		United States	Retrospective	8146	72	2013	Consecutive	Any
Stivanello et al.	2014	Weekday		Weekend		Italy	Retrospective	213539	36	2005-2010	Consecutive	Any
Palmer et al.	2015	Weekend		Weekday		United Kingdom	Retrospective	1332835	Not reported	2010-2012	Consecutive	Any
Kopec-Godlewska et	2018	Weekday		Weekend		Poland	Retrospective	88070	29	2013-2014	Consecutive	Any
Mirabal-Beltran et al.	2020	Weekend		Weekday		United States	Retrospective	1800	1	2010-2016	Consecutive	Any
Takegata et al.	2020	Weekend		Weekday		Vietnam	Prospective	3148	4	2016	Random	Any
**Weekday vs Sunday**												
Bertollini et al.	1992	Weekday		Sunday		Italy	Retrospective	136666	96	1985-1987	Consecutive	Any
Gomes et al. A	1999	Weekday		Sunday		Brazil	Prospective	6750	8	1978-1979	Consecutive	Any
Gomes et al. B	1999	Weekday		Sunday		Brazil	Prospective	2846	10	1994	Consecutive	Any
Campero et al.	2004	Sunday		Weekday		Mexico	Retrospective	992	Not reported	1987-2000	Consecutive	Any
Mossialos et al.	2005	Weekday		Sunday		Greece	Retrospective	805	3	2002	Consecutive	Any
de Almeida et al.	2008	Weekday		Sunday		Brazil	Retrospective	5800	2	1999	Consecutive	Any
Barros et al.	2011	Weekday		Sunday		Brazil	Prospective	4126	5	2004	Consecutive	Any
**Office hours vs out-of-office hours**												
Hoxha et al.	2019	Office hours	07:00 to 15:00 and weekday	Out of office hour	15:01 to 06:59 and weekend	Kosovo	Prospective	859	5	2015	Consecutive	Any
**Emergency CS**												
**Day vs night**												
Fraser et al.	1987	Day	08:00 to 17:59	Night	00:00 to 07:59	Canada	Retrospective	3169	1	1978-1984	Consecutive	Emergency
Goldstick et al.	2003	Night	16:00 to 07:59	Day	08:00 to 15:59	Israel	Retrospective	41626	1	1990-1998	Consecutive	Emergency
Bailit et al.	2006	Night	23:00 to 07:00	Day	07:00 to 15:00	United States	Prospective	11564	13	1999-2000	Consecutive	Emergency
Caughey et al.	2008	Night	00:00 to 07:00	Day	07:00 to 18:00	United States	Retrospective	28426	1	1976-2001	Consecutive	Emergency
Suzuki et al.	2010	Night	00.00 to 08.00	Day	08.00 to 16.00	Japan	Retrospective	8939	1	2002-2009	Consecutive	Emergency
Kalogiannidis et al.	2011	Night	23:00 to 07:00	Day	07:;00 to 23:00	Greece	Retrospective	3055	1	2004-2007	Consecutive	Emergency
Woodhead et al.	2012	Night	00:00 to 07:59	Day	08:00 to 15:59	United Kingdom	Retrospective	9820	Not reported	2004-2007	Consecutive	Emergency
Butler et al.	2014	Night	20:00 to 07:59	Day	08:00 to 19:59	Ireland	Prospective	597	1	2013	Consecutive	Emergency
Aiken et al.	2015	Day	n/a	Night	n/a	United Kingdom	Retrospective	868	1	2008-2013	Consecutive	Emergency
Brookfield et al.	2016	Night	00:00 to 06:59	Day	07:00 to 16:59	United States	Prospective	2641	13	2000-2001	Consecutive	Emergency
Sebastião et al.	2016	Day	08:00 to 15:59	Night	04:00 to 07:59	United States	Retrospective	236678	122	2004-2011	Consecutive	Emergency
Mgaya et al.	2017	Night	20:00 to 07:00	Day	07:00 to 14:00	Tanzania	Prospective	2059	1	2012	Consecutive	Emergency
Campillo-Artero et al	2018	Night	21:00 to 04:00	Day	04:00 to 21:00	Spain	Prospective	6157	4	2014	Consecutive	Emergency
Wehberg et al.	2018	Night	20:00 to 07:59	Day	08:00 to 19:59	Denmark	Retrospective	206992	29	2009-2012	Consecutive	Emergency
Eze et al.	2019	Night	12:00 to 19:59	Day	08:00 to 15:59	Nigeria	Prospective	2960	1	2016-2018	Consecutive	Emergency
Liu et al.	2019	Night	18:00 to 05:59	Day	06:00 to 17:59	United States	Retrospective	7691	1	2014-2015	Consecutive	Emergency
**Evening vs night**												
Fraser et al.	1987	Evening	18:00 to 00:00	Night	00:00 to 07:59	Canada	Retrospective	2087	1	1978-1984	Consecutive	Emergency
Sebastião et al.	2016	Evening	16:00 to 23:59	Night	04:00 to 07:59	United States	Retrospective	254656	122	2004-2011	Consecutive	Emergency
**Day vs evening**												
Bailit et al.	2006	Evening	15:00 to 23:00	Day	07:00 to 15:00	United States	Prospective	13631	13	1999-2000	Consecutive	Emergency
Caughey et al.	2008	Evening	18:00 to 23:59	Day	07:00 to 18:00	United States	Retrospective	22531	1	1976-2001	Consecutive	Emergency
Suzuki et al.	2010	Evening	16:00 to 24:00	Day	08.00 to 16.00	Japan	Retrospective	9653	1	2002-2009	Consecutive	Emergency
Woodhead et al.	2012	Evening	16:00 to 23:59	Day	08:00 to 15:59	United Kingdom	Retrospective	9186	Not reported	2004-2007	Consecutive	Emergency
Brookfield et al.	2016	Evening	17:00 to 23:59	Day	07:00 to 16:59	United States	Prospective	2704	13	2000-2001	Consecutive	Emergency
Mgaya et al.	2017	Evening	14:00 to 20:00	Day	07:00 to 14:00	Tanzania	Prospective	1357	1	2012	Consecutive	Emergency
Eze et al.	2019	Evening	16:00 to 11:59	Day	08:00 to 15:59	Nigeria	Prospective	3265	1	2016-2018	Consecutive	Emergency
**Weekday vs weekend**												
Fraser et al.	1987	Weekday		Weekend		Canada	Retrospective	4232	1	1978-1984	Consecutive	Emergency
Palmer et al.	2015	Weekend		Weekday		United Kingdom	Retrospective	1332835	Not reported	2010-2012	Consecutive	Emergency
Bommarito et al.	2016	Weekend		Weekday		United States	Retrospective	8627090	1051	1998-2010	Random	Emergency
Sebastião et al.	2016	Weekday		Weekend		United States	Retrospective	412192	122	2004-2011	Consecutive	Emergency
Wehberg et al.	2018	Weekend		Weekday		Denmark	Retrospective	206992	29	2009-2012	Consecutive	Emergency
Zbiri et al.	2018	Weekend	Weekend/Holiday delivery	Weekday	Working day	France	Retrospective	102236	11	2008-2014	Consecutive	Emergency
del Carmen et al.	2019	Weekend		Weekday		United States	Retrospective	1901352	Not reported	2006-2010	Consecutive	Emergency
Eze et al.	2019	Weekend		Weekday		Nigeria	Prospective	4556	1	2016-2018	Consecutive	Emergency
**Weekday vs sunday**												
Fabbri et al.	2016	Sunday		Weekday		Italy	Retrospective	8,908	Not reported	2007-2011	Random	Emergency
**Office hours vs out-of-office hours**												
Knight et al.	2016	Out of office hour	08:00 to 18:30	Office hours	18:30 to 08:00 and weekends or holidays	United Kingdom	Retrospective	83367	19	2012-2013	Consecutive	Emergency

		Type of data used							Robson criteria																
Author	Year	Survey	Hospital records	Birth certificates/registry	Claims data	Other	Source population	Source population by risk	Parity	Previous CS	Number of fetuses	Fetal presentation or lie	Gestational age	Onset of labour (induction)	Robson groups included in the study population										Categorization by Robson groups
**Any CS**																									
**Day vs night**																									
Hall et al.	1982		x				All births in Saint Josephs Hospital in Denver, Colorado, United States	Any risk	Any	Any	Any	Any	Any	Any	1	2	3	4	5	6	7	8	9	10	All
Busowski et al.	1997		x				All births in Department of Obstetrics and Gynecology of the University of South Florida, United States	Any risk	Any	Any	Any	Any	Any	Any	1	2	3	4	5	6	7	8	9	10	All
Gomes et al. B	1999	x					All births in Ribeirão Preto, State of São Paulo, Southeast Brazil	Any risk	Any	Any	1	Any	Any	Any	1	2	3	4	5	6	7		9	10	Multiple
Mitler et al.	2000		x				All births in 1 hospital in New Haven, Connecticut, United States	Any risk	Any	No	Any	Any	Any	Any	1	2	3	4		6	7	8	9	10	Multiple
Lee et al.	2003		x				All births in 17 hospitals, Canada	Extreme preterm	Any	Any	Any	Any	Any	Any	1	2	3	4	5	6	7	8	9	10	All
Campero et al.	2004		x				All births in Mexico City, Mexico	Any risk	Any	Any	Any	Any	Any	Any	1	2	3	4	5	6	7	8	9	10	All
Gould et al.	2005			x			All births in California hospitals	Any risk	Any	Any	Any	Any	Any	Any	1	2	3	4	5	6	7	8	9	10	All
Mossialos et al.	2005		x				All births in three hospitals, Athens, Greece	Any risk	Any	Any	Any	Any	Any	Any	1	2	3	4	5	6	7	8	9	10	All
D Orsi et al.	2006	x	x				All births in a public maternity hospital in the city of Rio de Janeiro, Brazil	Any risk	Any	Any	Any	Any	Any	Any	1	2	3	4	5	6	7	8	9	10	All
de Almeida et al.	2008		x	x			All births from two maternity clinics in Ribeirão Preto, São Paulo State, Brazil	Any risk	Any	Any	1	Any	Any	Any	1	2	3	4	5	6	7		9	10	Multiple
Bell et al.	2010					x	All births in 17 delivery centers, United States	VLBW	Any	Any	Any	Any	Any	Any	1	2	3	4	5	6	7	8	9	10	All
Osava et al.	2011		x				All births in 1 hospital in Sao Paolo, Brazil	Any risk	Any	Any	Any	Any	Any	Any	1	2	3	4	5	6	7	8	9	10	All
Caceres et al.	2013		x	x			All births in Massachusetts, United States	Any risk	0	No	1	Cephalic	>=37	Any	1	2									1 to 5
Stivanello et al.	2014		x	x			All births in Emilia-Romagna Region, Italy	Any risk	Any	Any	Any	Any	Any	Any	1	2	3	4	5	6	7	8	9	10	All
Brookfield et al.	2016		x				All births in 13 obstetric centers in United States	Any risk	Any	Any	1	Any	>=37	Any	1	2	3	4	5	6	7		9		Multiple
Frank-Wolf et al.	2016		x				All births in 1 hospital in Israel	Any risk	Any	Any	1	Any	Any	Any	1	2	3	4	5	6	7		9	10	Multiple
Saraiva et al.	2017		x				All births in 1 hospital in Porto Alegre, Brazil	Any risk	Any	Any	Any	Any	Any	Any	1	2	3	4	5	6	7	8	9	10	All
Mirabal-Beltran et al.	2020		x				All births at Medstar Washington Hospital Center (Hospital Center) in Washington DC, United States	Any risk	Any	Yes	1	Cephalic	>=37	Any					5						1 to 5
**Evening vs night**																									
Hall et al.	1982		x				All births in Saint Josephs Hospital in Denver, Colorado, United States	Any risk	Any	Any	Any	Any	Any	Any	1	2	3	4	5	6	7	8	9	10	All
Burns et al.	1995		x	x			All births in Arizona, United States	Any risk	Any	Any	Any	Any	Any	Any	1	2	3	4	5	6	7	8	9	10	All
Busowski et al.	1997		x				All births in Department of Obstetrics and Gynecology of the University of South Florida, United States	Any risk	Any	Any	Any	Any	Any	Any	1	2	3	4	5	6	7	8	9	10	All
Gomes et al. B	1999	x					All births in Ribeirão Preto, State of São Paulo, Southeast Brazil	Any risk	Any	Any	1	Any	Any	Any	1	2	3	4	5	6	7		9	10	Multiple
Campero et al.	2004		x				All births in Mexico City, Mexico	Any risk	Any	Any	Any	Any	Any	Any	1	2	3	4	5	6	7	8	9	10	All
Mossialos et al.	2005		x				All births in three hospitals, Athens, Greece	Any risk	Any	Any	Any	Any	Any	Any	1	2	3	4	5	6	7	8	9	10	All
D Orsi et al.	2006	x	x				All births in a public maternity hospital in the city of Rio de Janeiro, Brazil	Any risk	Any	Any	Any	Any	Any	Any	1	2	3	4	5	6	7	8	9	10	All
de Almeida et al.	2008		x	x			All births from two maternity clinics in Ribeirão Preto, São Paulo State, Brazil	Any risk	Any	Any	1	Any	Any	Any	1	2	3	4	5	6	7		9	10	Multiple
Frank-Wolf et al.	2016		x				All births in 1 hospital in Israel	Any risk	Any	Any	1	Any	Any	Any	1	2	3	4	5	6	7		9	10	Multiple
**Day and evening vs night**																									
Freitas et al.	2016		x			x	All births in 61 hospitals in Santa Catarina State, Brazil	Any risk	Any	Any	1	Any	Any	Any	1	2	3	4	5	6	7		9	10	Multiple
**Day vs evening**																									
Burns et al.	1995		x	x			All births in Arizona, United States	Any risk	Any	Any	Any	Any	Any	Any	1	2	3	4	5	6	7	8	9	10	All
Bell et al.	2010					x	All births in 17 delivery centers, United States	VLBW	Any	Any	Any	Any	Any	Any	1	2	3	4	5	6	7	8	9	10	All
Osava et al.	2011		x				All births in 1 hospital in Sao Paolo, Brazil	Any risk	Any	Any	Any	Any	Any	Any	1	2	3	4	5	6	7	8	9	10	All
Brookfield et al.	2016		x				All births in 13 obstetric centers in United States	Any risk	Any	Any	1	Any	>=37	Any	1	2	3	4	5	6	7		9		Multiple
Saraiva et al.	2017		x				All births in 1 hospital in Porto Alegre, Brazil	Any risk	Any	Any	Any	Any	Any	Any	1	2	3	4	5	6	7	8	9	10	All
**Weekday vs weekend**																									
Burns et al.	1995		x	x			All births in Arizona, United States	Any risk	Any	Any	Any	Any	Any	Any	1	2	3	4	5	6	7	8	9	10	All
Librero et al.	2000		x				All births in 11 public hospitals in Valencio, Spain	Any risk	Any	Any	Any	Any	Any	Any	1	2	3	4	5	6	7	8	9	10	All
Gould et al.	2003		x			x	All births in California, United States	Any risk	Any	Any	Any	Any	Any	Any	1	2	3	4	5	6	7	8	9	10	All
Tamim et al.	2007	x	x				All births in control hospitals (out of 9 hospitals) in the Greater Beirut area, Lebanon	Any risk	0	No	Any	Any	Any	Any	1	2				6		8	9	10	Multiple
Bell et al.	2010					x	All births in 17 delivery centers, United States	VLBW	Any	Any	Any	Any	Any	Any	1	2	3	4	5	6	7	8	9	10	All
Grytten et al.	2011			x			All births in Norway	Any risk	Any	Any	Any	Any	Any	Any	1	2	3	4	5	6	7	8	9	10	All
Marquez-Calderon et	2011					x	All birth in Andalusia, Spain	Any risk	Any	Any	Any	Any	Any	Any	1	2	3	4	5	6	7	8	9	10	All
Caceres et al.	2013		x	x			All births in Massachusetts, United States	Any risk	0	No	1	Cephalic	>=37	Any	1	2									1 to 5
Clark et al.	2014		x			x	All births in 72 hospitals in 16 states of Unites States	Any risk	0	No	Any	Any	>=37	Any	1	2				6		8	9		Multiple
Stivanello et al.	2014		x	x			All births in Emilia-Romagna Region, Italy	Any risk	Any	Any	Any	Any	Any	Any	1	2	3	4	5	6	7	8	9	10	All
Palmer et al.	2015				x		All births in England, United Kingdom	Any risk	Any	Any	Any	Any	Any	Any	1	2	3	4	5	6	7	8	9	10	All
Kopec-Godlewska et	2018					x	All births in 29 hospitals in Malopolska, Poland	Any risk	Any	Any	Any	Any	Any	Any	1	2	3	4	5	6	7	8	9	10	All
Mirabal-Beltran et al.	2020		x				All births at Medstar Washington Hospital Center (Hospital Center) in Washington DC, United States	Any risk	Any	Yes	1	Cephalic	>=37	Any					5						1 to 5
Takegata et al.	2020	x					All births in Nha Trang city	Any risk	Any	Any	Any	Any	Any	Any	1	2	3	4	5	6	7	8	9	10	All
**Weekday vs Sunday**																									
Bertollini et al.	1992			x			All births in Rome, Italy	Any risk	Any	No	1	Any	Any	Any	1	2	3	4		6	7		9	10	Multiple
Gomes et al. A	1999	x					All births in Ribeirão Preto, State of São Paulo, Southeast Brazil	Any risk	Any	Any	1	Any	Any	Any	1	2	3	4	5	6	7		9	10	Multiple
Gomes et al. B	1999	x					All births in Ribeirão Preto, State of São Paulo, Southeast Brazil	Any risk	Any	Any	1	Any	Any	Any	1	2	3	4	5	6	7		9	10	Multiple
Campero et al.	2004		x				All births in Mexico City, Mexico	Any risk	Any	Any	Any	Any	Any	Any	1	2	3	4	5	6	7	8	9	10	All
Mossialos et al.	2005		x				All births in three hospitals, Athens, Greece	Any risk	Any	Any	Any	Any	Any	Any	1	2	3	4	5	6	7	8	9	10	All
de Almeida et al.	2008		x	x			All births from two maternity clinics in Ribeirão Preto, São Paulo State, Brazil	Any risk	Any	Any	1	Any	Any	Any	1	2	3	4	5	6	7		9	10	Multiple
Barros et al.	2011	x	x				All births from urban area in Pelotas, Brazil	Any risk	Any	Any	1	Any	Any	Any	1	2	3	4	5	6	7		9	10	Multiple
**Office hours vs out-of-office hours**																									
Hoxha et al.	2019	x	x				All births in 5 hospitals, Kosovo	Any risk	0	No	Any	Cephalic	>=37	Any	1	2									1 to 5
**Emergency CS**																									
**Day vs night**																									
Fraser et al.	1987		x				All birth in the Royal Victoria Hospital,Canada	Dystocia	0	No	1	Any	>=37	Spontaneous	1										1 and 3
Goldstick et al.	2003		x				All births in 1 hospital in Haifa, Israel	Any risk	Any	Any	Any	Any	Any	Any	1	2	3	4	5	6	7	8	9	10	All
Bailit et al.	2006		x				All births in 13 tertiary hospitals in United States	Any risk	Any	Any	Any	Any	>=37	Any	1	2	3	4	5	6	7	8	9		Multiple
Caughey et al.	2008		x				All births in 1 hospital in San Francisco, California, United States	Any risk	Any	Any	1	Cephalic	>=37	Any	1	2	3	4	5						1 to 5
Suzuki et al.	2010		x				All births in 1 hospital in Tokyo, Japan	Any risk	Any	Any	1	Any	Any	Any	1	2	3	4	5	6	7		9	10	Multiple
Kalogiannidis et al.	2011		x				All births in the 4th Department of Obstetrics and Gynecology of Aristotle University of Thessaloniki, Greece	Any risk	Any	Any	1	Cephalic	>=37	Any	1	2	3	4	5						1 to 5
Woodhead et al.	2012		x	x			All births in Hull and East Yorkshire, England, United Kingdom	Any risk	Any	Any	Any	Any	Any	Any	1	2	3	4	5	6	7	8	9	10	All
Butler et al.	2014		x				All births in 1 hospital in Dublin, Ireland	Any risk	0	No	1	Cephalic	>=37	Any	1	2									1 to 5
Aiken et al.	2015		x				All births in single tertiary obstetrics center, United Kingdom	Malposition	Any	Any	1	Cephalic	>=37	Any	1	2	3	4	5						1 to 5
Brookfield et al.	2016		x				All births in 13 obstetric centers in United States	Any risk	Any	Any	1	Any	>=37	Any	1	2	3	4	5	6	7		9		Multiple
Sebastião et al.	2016		x	x			All births in 122 hospitals in Florida, United States	Any risk	0	No	1	Cephalic	>=37	Spontaneous	1										1 and 3
Mgaya et al.	2017	x	x				All births in 1 hospital in Dar es Salaam, Tanzania	Any risk	Any	Any	1	Any	Any	Any	1	2	3	4	5	6	7		9	10	Multiple
Campillo-Artero et al	2018					x	All births in 4 hospitals, Spain	Any risk	Any	Any	1	Any	Any	Any	1	2	3	4	5	6	7		9	10	Multiple
Wehberg et al.	2018			x			All births in 29 maternity units in Denmark	Any risk	Any	Any	1	Any	Any	Any	1	2	3	4	5	6	7		9	10	Multiple
Eze et al.	2019	x	x	x			All births in 1 hospital in Abakaliki, Nigeria	GDM*	Any	Any	1	Any	>=37	Any	1	2	3	4	5	6	7		9		Multiple
Liu et al.	2019		x				All births in 1 hospital in United States	Any risk	0	No	1	Cephalic	>=37	Any	1	2									1 to 5
**Evening vs night**																									
Fraser et al.	1987		x				All birth in the Royal Victoria Hospital,Canada	Dystocia	0	No	1	Any	>=37	Spontaneous	1										1 and 3
Sebastião et al.	2016		x	x			All births in 122 hospitals in Florida, United States	Any risk	0	No	1	Cephalic	>=37	Spontaneous	1										1 and 3
**Day vs evening**																									
Bailit et al.	2006		x				All births in 13 tertiary hospitals in United States	Any risk	Any	Any	Any	Any	>=37	Any	1	2	3	4	5	6	7	8	9		Multiple
Caughey et al.	2008		x				All births in 1 hospital in San Francisco, California, United States	Any risk	Any	Any	1	Cephalic	>=37	Any	1	2	3	4	5						1 to 5
Suzuki et al.	2010		x				All births in 1 hospital in Tokyo, Japan	Any risk	Any	Any	1	Any	Any	Any	1	2	3	4	5	6	7		9	10	Multiple
Woodhead et al.	2012		x	x			All births in Hull and East Yorkshire, England, United Kingdom	Any risk	Any	Any	Any	Any	Any	Any	1	2	3	4	5	6	7	8	9	10	All
Brookfield et al.	2016		x				All births in 13 obstetric centers in United States	Any risk	Any	Any	1	Any	>=37	Any	1	2	3	4	5	6	7		9		Multiple
Mgaya et al.	2017	x	x				All births in 1 hospital in Dar es Salaam, Tanzania	Any risk	Any	Any	1	Any	Any	Any	1	2	3	4	5	6	7		9	10	Multiple
Eze et al.	2019	x	x	x			All births in 1 hospital in Abakaliki, Nigeria	GDM*	Any	Any	1	Any	>=37	Any	1	2	3	4	5	6	7		9		Multiple
**Weekday vs weekend**																									
Fraser et al.	1987		x				All birth in the Royal Victoria Hospital,Canada	Dystocia	0	No	1	Any	>=37	Spontaneous	1										1 and 3
Palmer et al.	2015				x		All births in England, United Kingdom	Any risk	Any	Any	Any	Any	Any	Any	1	2	3	4	5	6	7	8	9	10	All
Bommarito et al.	2016		x				All births in 1051 hospitals, United States	Any risk	Any	Any	Any	Any	Any	Any	1	2	3	4	5	6	7	8	9	10	All
Sebastião et al.	2016		x	x			All births in 122 hospitals in Florida, United States	Any risk	0	No	1	Cephalic	>=37	Spontaneous	1										1 and 3
Wehberg et al.	2018			x			All births in 29 maternity units in Denmark	Any risk	Any	Any	1	Any	Any	Any	1	2	3	4	5	6	7		9	10	Multiple
Zbiri et al.	2018			x		x	All births in 11 hospitals in Yveline, France	Any risk	Any	Any	Any	Any	Any	Any	1	2	3	4	5	6	7	8	9	10	All
del Carmen et al.	2019		x		x		All births of all hospitals in California, United States	Any risk	Any	No	1	Cephalic	>=37	Spontaneous	1		3								1 and 3
Eze et al.	2019	x	x	x			All births in 1 hospital in Abakaliki, Nigeria	GDM*	Any	Any	1	Any	>=37	Any	1	2	3	4	5	6	7		9		Multiple
**Weekday vs sunday**																									
Fabbri et al.	2016		x	x			All births in Ospedale Maggiore in Bologna, Italy	Any risk	Any	Any	1	Any	Any	Any	1	2	3	4	5	6	7		9	10	Multiple
**Office hours vs out-of-office hours**																									
Knight et al.	2016		x				All births in 19 hospitals, United Kingdom	Any risk	Any	Any	1	Any	Any	Any	1	2	3	4	5	6	7		9	10	Multiple

		Covariates used for statistical adjustment																						QUIPS risk of bias					
		Maternal preconception status									Maternal clinical status					Fetus characteristics													
Author	Year	Ethnicity/Race	Educational level	Marital status	Economic status	Insurances status	Urban status	Weight	Height	Body mass index	Age	Parity	Previous caesarean section	Pre-existing (before pregnancy) conditions	Conditions developed during pregnancy	Gestational age	Birth weight	Other characteristics	Prenatal care	Birth characteristics	Provider characteristics	Other variables	Total number of covariates	Study Participation	Study Attrition	Prognostic Factor Measurement	Outcome Measurement	Study Confounding	Statistical Analysis and Reporting
**Any CS**																													
**Day vs night**																													
Hall et al.	1982																						0	low	low	low	low	high	low
Busowski et al.	1997																						0	low	low	low	low	high	low
Gomes et al. B	1999		x		x	x													x		xx	xx	9	low	low	low	low	moderate	low
Mitler et al.	2000	x									x	x			xx		x	xx			xx	xx	18	low	low	low	low	low	low
Lee et al.	2003																						0	low	low	low	low	high	low
Campero et al.	2004		x								x		x				x	x	x	x	xx	xx	12	low	low	low	low	moderate	low
Gould et al.	2005																						0	low	low	low	low	moderate	low
Mossialos et al.	2005	x			x	x																xx	3	moderate	low	low	low	moderate	low
D Orsi et al.	2006		x								x		x		x	x		x	x	xx	xx	xx	22	moderate	low	low	low	low	low
de Almeida et al.	2008																						0	low	low	low	low	high	low
Bell et al.	2010																						0	low	low	low	low	high	low
Osava et al.	2011																						0	low	low	low	low	high	low
Caceres et al.	2013	x	x								x			xx	xx	x	x			x			10	low	low	low	low	low	low
Stivanello et al.	2014																						0	low	low	low	low	high	low
Brookfield et al.	2016																						0	low	low	low	low	high	low
Frank-Wolf et al.	2016																						0	low	low	low	low	high	low
Saraiva et al.	2017																						0	low	low	low	low	high	low
Mirabal-Beltran et al.	2020	x	x	x					x	x	x	x			x	x	x			x	xx	xx	17	low	low	low	low	low	low
**Evening vs night**																													
Hall et al.	1982																						0	low	low	low	low	high	low
Burns et al.	1995	x	x								x	x	x		xx	x	x	x	x		xx	xx	33	low	low	low	low	low	low
Busowski et al.	1997																						0	low	low	low	low	high	low
Gomes et al. B	1999		x		x	x													x		xx	xx	9	low	low	low	low	moderate	low
Campero et al.	2004		x								x		x				x	x	x	x	xx	xx	12	low	low	low	low	moderate	low
Mossialos et al.	2005	x			x	x																xx	3	moderate	low	low	low	moderate	low
D Orsi et al.	2006		x								x		x		x	x		x	x	xx	xx	xx	22	moderate	low	low	low	low	low
de Almeida et al.	2008										x	x		x							x	xx	6	low	low	low	low	high	low
Frank-Wolf et al.	2016																						0	low	low	low	low	high	low
**Day and evening vs night**																													
Freitas et al.	2016	x	x	x							x	x	x			x			x		x	x	10	low	low	low	low	high	low
**Day vs evening**																													
Burns et al.	1995	x	x								x	x	x		xx	x	x	x	x		xx	xx	33	low	low	low	low	low	low
Bell et al.	2010																						0	low	low	low	low	high	low
Osava et al.	2011																						0	low	low	low	low	high	low
Brookfield et al.	2016																						0	low	low	low	low	high	low
Saraiva et al.	2017																						0	low	low	low	low	high	low
**Weekday vs weekend**																													
Burns et al.	1995	x	x								x	x	x		xx	x	x	x	x		xx	xx	33	low	low	low	low	low	low
Librero et al.	2000							x			x		x				x	xx				xx	10	low	low	low	low	low	low
Gould et al.	2003																						0	low	low	low	low	high	low
Tamim et al.	2007										x					x	x	x	x		x	xx	9	low	low	low	low	high	low
Bell et al.	2010																						0	low	low	low	low	high	low
Grytten et al.	2011																						24	low	low	low	low	low	low
Marquez-Calderon et	2011	x	x	x							x	x					x					xx	8	low	low	low	low	moderate	low
Caceres et al.	2013	x	x								x			xx	xx	x	x			x			10	low	low	low	low	low	low
Clark et al.	2014																						0	low	low	low	low	high	low
Stivanello et al.	2014																						0	low	low	low	low	high	low
Palmer et al.	2015																						0	low	low	low	low	high	low
Kopec-Godlewska et	2018																						0	low	low	low	low	high	low
Mirabal-Beltran et al.	2020	x	x	x					x	x	x	x			x	x	x			x	xx	xx	17	low	low	low	low	low	low
Takegata et al.	2020																						0	low	low	low	low	moderate	low
**Weekday vs Sunday**																													
Bertollini et al.	1992										x	x				x	x			x		x	6	low	low	low	low	moderate	low
Gomes et al. A	1999		x		x	x													x		x	xx	7	low	low	low	low	moderate	low
Gomes et al. B	1999		x		x	x													x		xx	xx	9	low	low	low	low	moderate	low
Campero et al.	2004		x								x		x				x	x	x	x	xx	xx	12	low	low	low	low	moderate	low
Mossialos et al.	2005	x			x	x																xx	3	moderate	low	low	low	moderate	low
de Almeida et al.	2008										x	x		x							xx	xx	7	low	low	low	low	high	low
Barros et al.	2011																						0	low	low	low	low	high	low
**Office hours vs out-of-office hours**																													
Hoxha et al.	2019		x		x	x	x				x								x		xx	xx	12	moderate	low	low	low	low	low
**Emergency CS**																													
**Day vs night**																													
Fraser et al.	1987																						0	low	low	low	low	high	low
Goldstick et al.	2003																						0	low	low	low	low	high	low
Bailit et al.	2006																						0	low	low	low	low	high	low
Caughey et al.	2008																						0	low	low	low	low	high	low
Suzuki et al.	2010																						0	low	low	low	low	high	low
Kalogiannidis et al.	2011																						0	low	low	low	low	high	low
Woodhead et al.	2012																						0	low	low	low	low	high	low
Butler et al.	2014																						0	low	low	low	low	high	low
Aiken et al.	2015																						0	low	low	low	low	high	low
Brookfield et al.	2016																						0	low	low	low	low	high	low
Sebastião et al.	2016	x		x		x				x	x			x		x				x	x	x	10	low	low	low	low	low	low
Mgaya et al.	2017																						0	low	low	low	low	low	low
Campillo-Artero et al	2018									x	x	x	x							x		xx	14?	low	low	low	low	low	low
Wehberg et al.	2018								x	x	x	x		xx	xx	x		xx		x		xx	18	low	low	low	low	low	low
Eze et al.	2019																						0	low	low	low	low	high	low
Liu et al.	2019																						0	low	low	low	low	high	low
**Evening vs night**																													
Fraser et al.	1987																						0	low	low	low	low	high	low
Sebastião et al.	2016	x		x		x				x	x			x		x				x	x	x	10	low	low	low	low	low	low
**Day vs evening**																													
Bailit et al.	2006																						0	low	low	low	low	high	low
Caughey et al.	2008																						0	low	low	low	low	high	low
Suzuki et al.	2010																						0	low	low	low	low	high	low
Woodhead et al.	2012																						0	low	low	low	low	high	low
Brookfield et al.	2016																						0	low	low	low	low	high	low
Mgaya et al.	2017																						0	low	low	low	low	high	low
Eze et al.	2019																						0	low	low	low	low	high	low
**Weekday vs weekend**																													
Fraser et al.	1987																						0	low	low	low	low	high	low
Palmer et al.	2015																						0	low	low	low	low	high	low
Bommarito et al.	2016					x	x				x		x	xx	xx			xx		xx	xx	xx	?	low	low	low	low	high	low
Sebastião et al.	2016	x		x		x				x	x			x		x				x	x	x	10	low	low	low	low	low	low
Wehberg et al.	2018								x	x	x	x		xx	xx	x		xx		x		xx	18	low	low	low	low	low	low
Zbiri et al.	2018										x	x	x				x	x		xx	xx	xx	17	low	low	low	low	low	low
del Carmen et al.	2019	x				x	x				x			xx	xx			x		x	xx	xx	16	low	low	low	low	low	low
Eze et al.	2019																						0	low	low	low	low	high	low
**Weekday vs sunday**																													
Fabbri et al.	2016																						0	low	low	low	low	low	low
**Office hours vs out-of-office hours**																													
Knight et al.	2016																				xx		xx	7	low	low	low	low	moderate	low

Subgroup analysis of adjusted estimates: Any CS, Weekday vs weekend

Category and Subgroups	Number of studies	OR (95% CI)	τ^2	p for interaction
**Country**				
Lebanon	1	1.38 (1.16, 1.64)	.	0.459
Norway	1	1.64 (1.32, 2.04)	.	.
Spain	2	1.50 (1.36, 1.64)	0.003	.
United States	3	1.27 (0.95, 1.70)	0.057	.
**Period of data collection**				
2000 and after	4	1.36 (0.99, 1.87)	0.096	0.298
Before 2000	2	1.37 (1.26, 1.48)	<0.001	.
Before and after 2000	1	1.64 (1.32, 2.04)	.	.
**CS rate**				
19% or less	1	1.40 (1.25, 1.57)	.	0.387
20%-40%	2	1.51 (1.38, 1.66)	0.003	.
41% and more	1	1.72 (1.27, 2.35)	.	.
Not reported	3	1.27 (0.95, 1.70)	0.061	.
**Population by Robson**				
1 to 5	2	1.27 (0.73, 2.21)	0.147	0.746
All	4	1.47 (1.34, 1.60)	0.005	.
Multiple	1	1.38 (1.16, 1.64)	.	.
**Parity**				
0	2	1.15 (0.82, 1.61)	0.054	0.150
Any	5	1.48 (1.36, 1.61)	0.005	.
**Inclusion of women with previous CS**				
Any	4	1.47 (1.34, 1.60)	0.005	0.216
No	2	1.15 (0.82, 1.61)	0.054	.
Yes	1	1.72 (1.27, 2.35)	.	.
**Number of fetuses**				
1	2	1.27 (0.73, 2.21)	0.147	0.636
Any	5	1.46 (1.34, 1.58)	0.005	.
**Fetal presentation**				
Any	5	1.46 (1.34, 1.58)	0.005	0.636
Cephalic	2	1.27 (0.73, 2.21)	0.147	.
**Gestational age**				
>=37	2	1.27 (0.73, 2.21)	0.147	0.636
Any	5	1.46 (1.34, 1.58)	0.005	.
**QUIPS confounding**				
Low	6	1.36 (1.11, 1.67)	0.056	0.220
Moderate	1	1.55 (1.51, 1.59)	.	.
**Hospital data**				
Yes	5	1.32 (1.07, 1.63)	0.051	0.133
No	2	1.55 (1.51, 1.59)	<0.001	.
**Birth registry data**				
No	4	1.49 (1.39, 1.61)	0.002	0.291
Yes	3	1.27 (0.95, 1.70)	0.061	.

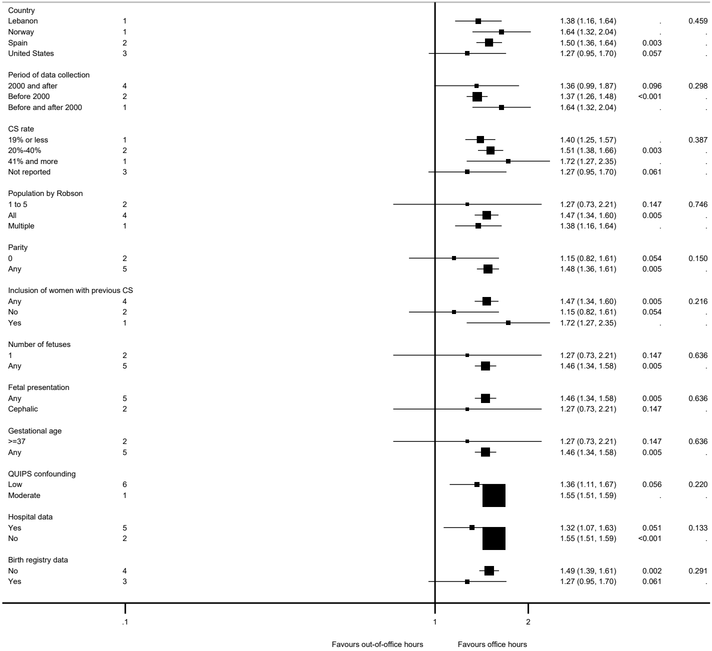

Subgroup analysis of adjusted estimates: Any CS, Weekday vs Sunday

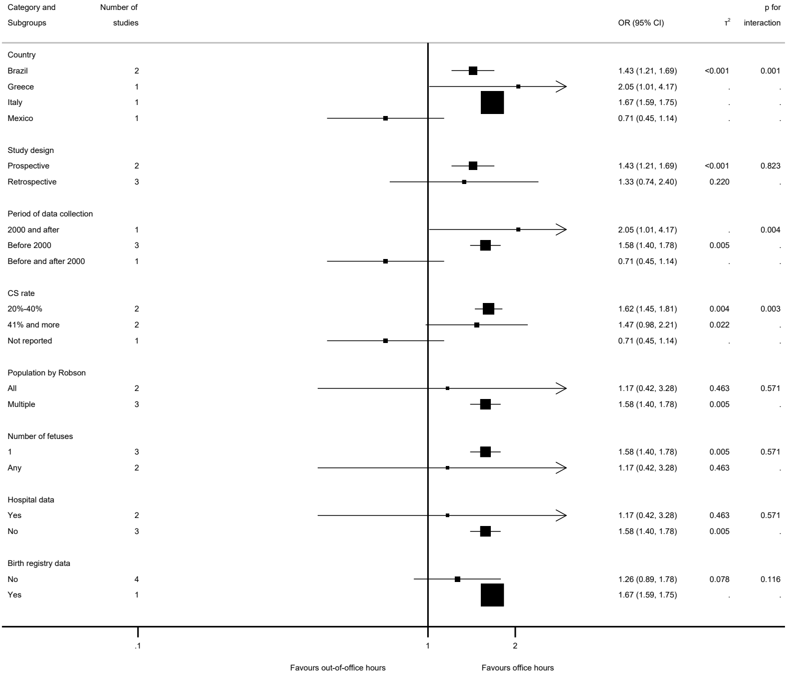

Descriptive presentation of adjusted estimates of other subgroups of studies: Any CS

Subgroup and Study	Year	Country	Sample size	Study design	Period of data collection	CS rate	Population by Robson	Source population	Women with existing conditions	Conditions developed during delivery	Odds Ratio (95% CI)
Day vs night											
Gomes et al. B	1999	Brazil	2846	Prospective	Before 2000	47.23	Multiple	Any risk	Included	Included	3.41 (2.38, 4.89)
Mitler et al.	2000	United States	1000	Retrospective	Before 2000	49.90	Multiple	Any risk	Included	Included	1.33 (0.99, 1.80)
Campero et al.	2004	Mexico	669	Retrospective	Before and after 2000	33.48	All	Any risk	Included	Included	2.20 (1.48, 3.27)
Mossialos et al.	2005	Greece	612	Retrospective	2000 and after	48.69	All	Any risk	Included	Included	3.45 (1.95, 6.09)
D Orsi et al.	2006	Brazil	231	Retrospective	Before 2000	53.25	All	Any risk	Included	Included	2.70 (0.88, 8.25)
Caceres et al.	2013	United States	54299	Retrospective	2000 and after	34.28	1 to 5	Any risk	Included	Included	1.07 (1.03, 1.12)
Mirabal-Beltran et al.	2020	United States	1800	Retrospective	2000 and after	73.40	1 to 5	Any risk	Included	Included	1.72 (1.34, 2.23)
Evening vs night											
Burns et al.	1995	United States	33233	Retrospective	Before 2000		All	Any risk	Included	Included	1.46 (1.27, 1.68)
Gomes et al. B	1999	Brazil	2846	Prospective	Before 2000	47.32	Multiple	Any risk	Included	Included	4.65 (3.13, 6.92)
Campero et al.	2004	Mexico	622	Retrospective	Before and after 2000	26.85	All	Any risk	Included	Included	1.50 (0.99, 2.27)
Mossialos et al.	2005	Greece	319	Retrospective	2000 and after	35.11	All	Any risk	Included	Included	2.25 (1.18, 4.29)
D Orsi et al.	2006	Brazil	266	Retrospective	Before 2000	47.74	All	Any risk	Included	Included	1.40 (0.48, 4.06)
Day vs evening											
Burns et al.	1995	United States	33233	Retrospective	Before 2000		All	Any risk	Included	Included	1.35 (1.22, 1.49)
Office hours vs out of office hours											
Hoxha et al.	2019	Kosovo	859	Prospective	2000 and after	20.72	1 to 5	Any risk	Excluded	Excluded	2.36 (1.37, 4.06)

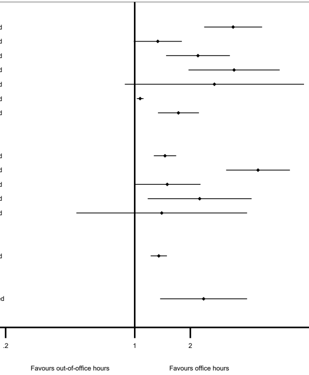

Descriptive presentation of crude estimates of other subgroups of studies: Any CS

Subgroup and Study	Year	Country	Sample size	Study design	Period of data collection	CS rate	Population by Robson	Source population	Women with existing conditions	Conditions developed during delivery	Odds Ratio (95% CI)
Day vs night											
Hall et al.	1982	United States	878	Prospective	Before 2000	15.77	All	Any risk	Included	Included	2.25 (1.34, 3.79)
Busowski et al.	1997	United States	3449	Retrospective	Before 2000	35.81	All	Any risk	Included	Included	3.16 (2.64, 3.79)
Gomes et al. B	1999	Brazil	2846	Prospective	Before 2000	47.23	Multiple	Any risk	Included	Included	3.18 (2.53, 4.00)
Mitler et al.	2000	United States	1000	Retrospective	Before 2000	49.90	Multiple	Any risk	Included	Included	1.32 (1.03, 1.69)
Lee et al.	2003	Canada	5192	Retrospective	Before 2000	48.11	All	Extreme preterm	Included	Excluded	1.21 (1.08, 1.35)
Campero et al.	2004	Mexico	669	Retrospective	Before and after 2000	33.48	All	Any risk	Included	Included	2.60 (1.85, 3.66)
Gould et al.	2005	United States	3362890	Retrospective	Before 2000	20.97	All	Any risk	Included	Included	1.64 (1.63, 1.65)
Mossialos et al.	2005	Greece	612	Retrospective	2000 and after	48.69	All	Any risk	Included	Included	3.14 (1.84, 5.35)
D Orsi et al.	2006	Brazil	231	Retrospective	Before 2000	53.25	All	Any risk	Included	Included	2.76 (1.58, 4.81)
de Almeida et al.	2008	Brazil	4005	Retrospective	Before 2000	48.44	Multiple	Any risk	Included	Included	2.14 (1.70, 2.70)
Bell et al.	2010	United States	7559	Prospective	2000 and after	62.15	All	VLBW	Included	Excluded	1.69 (1.53, 1.88)
Osava et al.	2011	Brazil	1949	Retrospective	2000 and after	15.39	All	Any risk	Included	Included	1.85 (1.42, 2.42)
Caceres et al.	2013	United States	54299	Retrospective	2000 and after	34.28	1 to 5	Any risk	Included	Included	1.31 (1.27, 1.36)
Stivanello et al.	2014	Italy	213539	Retrospective	2000 and after	30.26	All	Any risk	Included	Included	2.42 (2.37, 2.47)
Brookfield et al.	2016	United States	2928	Prospective	2000 and after	22.17	Multiple	Any risk	Excluded	Excluded	1.69 (1.40, 2.05)
Frank-Wolf et al.	2016	Israel	37996	Retrospective	2000 and after	7.16	Multiple	Any risk	Included	Excluded	1.75 (1.61, 1.90)
Saraiva et al.	2017	Brazil	301	Prospective	2000 and after	29.24	All	Any risk	Included	Included	3.57 (1.84, 6.95)
Mirabal-Beltran et al.	2020	United States	1800	Retrospective	2000 and after	73.40	1 to 5	Any risk	Included	Included	2.63 (2.11, 3.27)
Evening vs night											
Hall et al.	1982	United States	467	Prospective	Before 2000	15.73	All	Any risk	Included	Included	2.76 (1.56, 4.88)
Busowski et al.	1997	United States	3478	Retrospective	Before 2000	25.39	All	Any risk	Included	Included	1.27 (1.06, 1.54)
Gomes et al. B	1999	Brazil	2846	Prospective	Before 2000	47.32	Multiple	Any risk	Included	Included	3.98 (3.10, 5.11)
Campero et al.	2004	Mexico	622	Retrospective	Before and after 2000	26.85	All	Any risk	Included	Included	1.60 (1.12, 2.29)
Mossialos et al.	2005	Greece	319	Retrospective	2000 and after	35.11	All	Any risk	Included	Included	1.91 (1.04, 3.50)
D Orsi et al.	2006	Brazil	266	Retrospective	Before 2000	47.74	All	Any risk	Included	Included	1.85 (1.09, 3.14)
de Almeida et al.	2008	Brazil	2792	Retrospective	Before 2000	48.21	Multiple	Any risk	Included	Included	2.08 (1.62, 2.66)
Frank-Wolf et al.	2016	Israel	36828	Retrospective	2000 and after	5.95	Multiple	Any risk	Included	Excluded	1.26 (1.16, 1.37)
Day and evening vs night											
Freitas et al.	2016	Brazil	60904	Retrospective	2000 and after	60.63	Multiple	Any risk	Included	Included	4.14 (3.93, 4.36)
Day vs evening											
Bell et al.	2010	United States	8009	Prospective	2000 and after	66.18	All	VLBW	Included	Excluded	1.12 (1.02, 1.24)
Osava et al.	2011	Brazil	1671	Retrospective	2000 and after	16.94	All	Any risk	Included	Included	1.45 (1.07, 1.96)
Brookfield et al.	2016	United States	3034	Prospective	2000 and after	23.10	Multiple	Any risk	Excluded	Excluded	1.37 (1.15, 1.64)
Saraiva et al.	2017	Brazil	266	Prospective	2000 and after	37.59	All	Any risk	Included	Included	0.80 (0.44, 1.44)
Office hours vs out of office hours											
Hoxha et al.	2019	Kosovo	859	Prospective	2000 and after	20.72	1 to 5	Any risk	Excluded	Excluded	2.81 (2.00, 3.94)

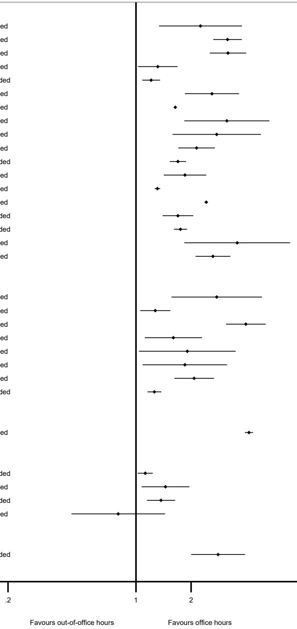

Subgroup analysis of adjusted estimates: Emergency CS, weekday vs weekend

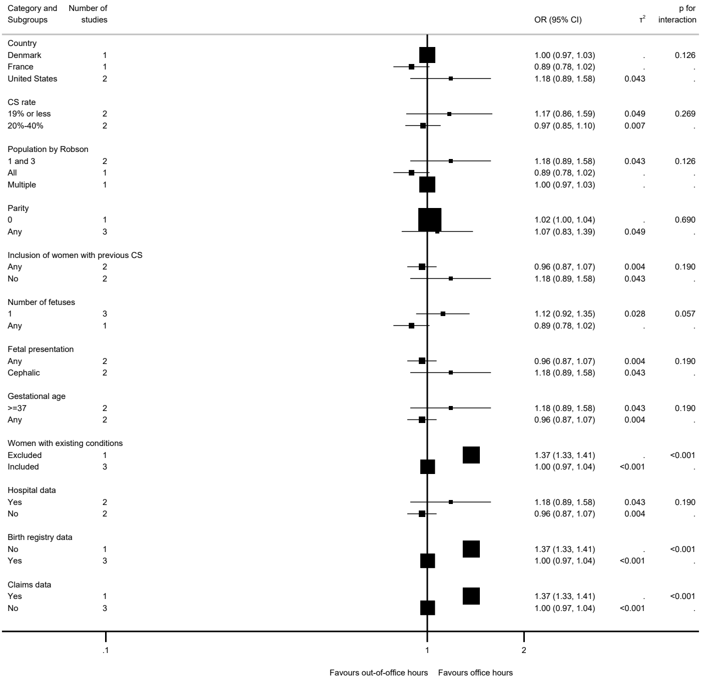

Descriptive presentation of adjusted estimates of other subgroups of studies: Emergency CS

Subgroup and Study	Year	Country	Sample size	Study design	Period of data collection	CS rate	Population by Robson	Source population	Women with existing conditions	Conditions developed during delivery	Odds Ratio (95% CI)
Day vs night											
Sebastião et al.	2016	United States	236678	Retrospective	2000 and after	20.86	1 and 3	Any risk	Included	Included	1.19 (1.16, 1.22)
Mgaya et al.	2017	Tanzania	2059	Prospective	2000 and after	46.96	Multiple	Any risk	Excluded	Excluded	1.00 (0.58, 1.73)
Campillo-Artero et al.	2018	Spain	6157	Prospective	2000 and after	11.00	Multiple	Any risk	Included	Included	0.67 (0.60, 0.75)
Wehberg et al.	2018	Denmark	206992	Retrospective	2000 and after	12.71	Multiple	Any risk	Included	Included	1.08 (1.05, 1.10)
Evening vs night											
Sebastião et al.	2016	United States	254656	Retrospective	2000 and after	24.75	1 and 3	Any risk	Included	Included	1.50 (1.46, 1.54)
Office hours vs out of office hours											
Knight et al.	2016	United Kingdom	83367	Retrospective	2000 and after	13.04	Multiple	Any risk	Included	Included	1.06 (1.02, 1.11)

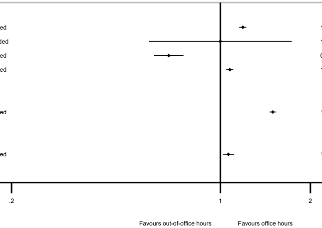

Descriptive presentation of crude estimates of other subgroups of studies: Emergency CS

Subgroup and Study	Year	Country	Sample size	Study design	Period of data collection	CS rate	Population by Robson	Source population	Women with existing conditions	Conditions developed during delivery	Odds Ratio (95% CI)
Day vs night											
Fraser et al.	1987	Canada	3169	Retrospective	Before 2000	3.79	1 and 3	Dystocia	Included	Included	0.95 (0.65, 1.39)
Goldstick et al.	2003	Israel	41626	Retrospective	Before 2000	12.71	All	Any risk	Included	Included	2.00 (1.88, 2.12)
Bailit et al.	2006	United States	11564	Prospective	Before 2000	4.48	Multiple	Any risk	Included	Included	1.39 (1.16, 1.66)
Caughey et al.	2008	United States	28426	Retrospective	Before and after 2000	12.32	1 to 5	Any risk	Included	Excluded	1.02 (0.95, 1.10)
Suzuki et al.	2010	Japan	8939	Retrospective	2000 and after	10.16	Multiple	Any risk	Included	Excluded	4.17 (3.46, 5.02)
Kalogiannidis et al.	2011	Greece	3055	Retrospective	2000 and after	9.75	1 to 5	Any risk	Included	Excluded	1.19 (0.90, 1.57)
Woodhead et al.	2012	United Kingdom	9820	Retrospective	2000 and after	14.82	All	Any risk	Included	Included	1.12 (1.00, 1.26)
Butler et al.	2014	Ireland	597	Prospective	2000 and after	7.37	1 to 5	Any risk	Included	Included	1.19 (0.64, 2.21)
Aiken et al.	2015	United Kingdom	868	Retrospective	2000 and after	61.52	1 to 5	Malposition	Included	Included	0.59 (0.45, 0.78)
Brookfield et al.	2016	United States	2641	Prospective	2000 and after	13.71	Multiple	Any risk	Excluded	Excluded	0.83 (0.66, 1.03)
Sebastião et al.	2016	United States	195268	Retrospective	2000 and after	21.36	1 and 3	Any risk	Included	Included	1.25 (1.22, 1.29)
Mgaya et al.	2017	Tanzania	2059	Prospective	2000 and after	46.96	Multiple	Any risk	Excluded	Excluded	0.95 (0.79, 1.14)
Wehberg et al.	2018	Denmark	206992	Retrospective	2000 and after	12.71	Multiple	Any risk	Included	Included	1.14 (1.10, 1.17)
Eze et al.	2019	Nigeria	2960	Prospective	2000 and after	18.82	Multiple	GDM*	Included	Excluded	1.11 (0.92, 1.34)
Liu et al.	2019	United States	7691	Retrospective	2000 and after	18.76	1 to 5	Any risk	Included	Included	0.79 (0.70, 0.88)
Evening vs night											
Fraser et al.	1987	Canada	2087	Retrospective	Before 2000	5.27	1 and 3	Dystocia	Included	Included	1.73 (1.16, 2.58)
Sebastião et al.	2016	United States	213246	Retrospective	2000 and after	25.97	1 and 3	Any risk	Included	Included	1.69 (1.65, 1.74)
Day vs evening											
Bailit et al.	2006	United States	13631	Prospective	Before 2000	4.94	Multiple	Any risk	Included	Included	1.08 (0.92, 1.26)
Caughey et al.	2008	United States	22531	Retrospective	Before and after 2000	12.67	1 to 5	Any risk	Included	Excluded	0.91 (0.83, 1.00)
Suzuki et al.	2010	Japan	9653	Retrospective	2000 and after	13.61	Multiple	Any risk	Included	Excluded	1.20 (1.07, 1.36)
Woodhead et al.	2012	United Kingdom	9186	Retrospective	2000 and after	15.18	All	Any risk	Included	Included	1.06 (0.95, 1.19)
Brookfield et al.	2016	United States	2704	Prospective	2000 and after	13.72	Multiple	Any risk	Excluded	Excluded	0.83 (0.66, 1.03)
Mgaya et al.	2017	Tanzania	1357	Prospective	2000 and after	46.43	Multiple	Any risk	Excluded	Excluded	0.99 (0.80, 1.23)
Eze et al.	2019	Nigeria	3265	Prospective	2000 and after	20.46	Multiple	GDM*	Included	Excluded	0.89 (0.75, 1.06)
Office hours vs out of office hours											
Knight et al.	2016	United Kingdom	83367	Retrospective	2000 and after	13.04	Multiple	Any risk	Included	Included	0.88 (0.85, 0.92)

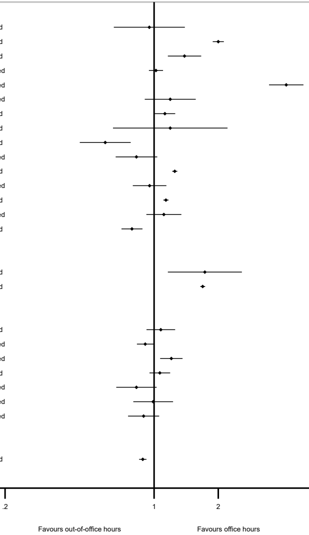

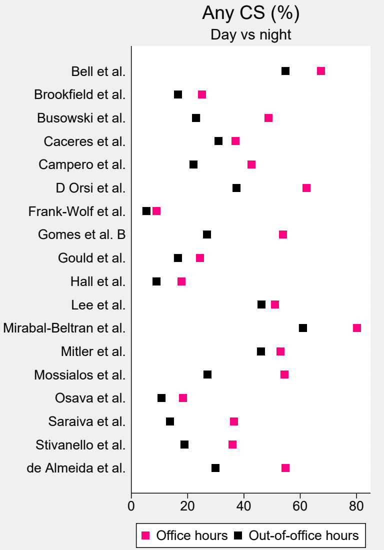
Any CS (%)
Day vs night
Bell et al.
Brookfield et al.
Busowski et al.
Caceres et al.
Campero et al.
D Orsi et al.
Frank-Wolf et al.
Gomes et al. B
Gould et al.
Hall et al.
Lee et al.
Mirabal-Beltran et al.
Mitler et al.
Mossialos et al.
Osava et al.
Saraiva et al.
Stivanello et al.
de Almeida et al.
0
20
40
60
80
Office hours
Out-of-office hours

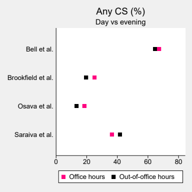

Any CS (%)
Day vs evening
Bell et al.
Brookfield et al.
Osava et al.
Saraiva et al.
0
20
40
60
80
Office hours
Out-of-office hours

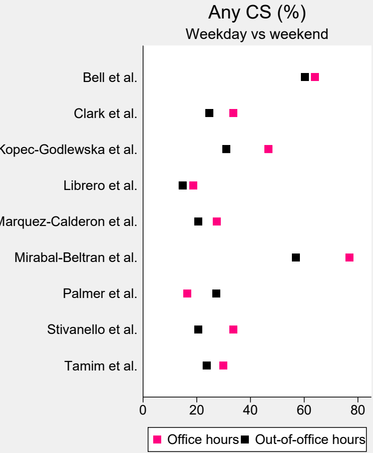

Any CS (%)
Weekday vs weekend
Bell et al.
Clark et al.
Kopec-Godlewska et al.
Librero et al.
Marquez-Calderon et al.
Mirabal-Beltran et al.
Palmer et al.
Stivanello et al.
Tamim et al.
0
20
40
60
80
Office hours
Out-of-office hours

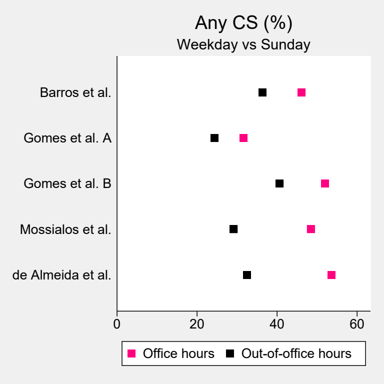

Any CS (%)
Weekday vs Sunday
Barros et al.
Gomes et al. A
Gomes et al. B
Mossialos et al.
de Almeida et al.
0
20
40
60
Office hours
Out-of-office hours

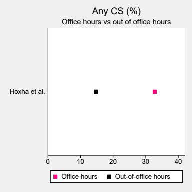

Any CS (%)
Office hours vs out of office hours
Hoxha et al.
0
10
20
30
40
Office hours
Out-of-office hours

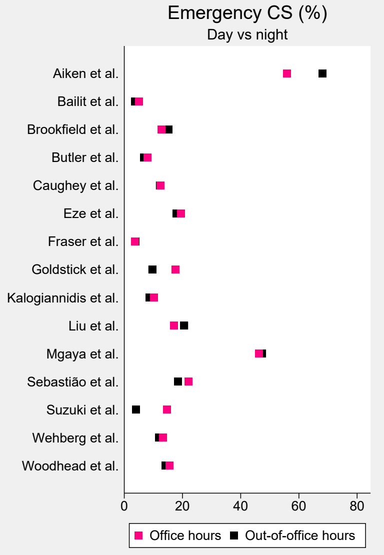
Emergency CS (%)
Day vs night
Aiken et al.
Bailit et al.
Brookfield et al.
Butler et al.
Caughey et al.
Eze et al.
Fraser et al.
Goldstick et al.
Kalogiannidis et al.
Liu et al.
Mgaya et al.
Sebastião et al.
Suzuki et al.
Wehberg et al.
Woodhead et al.
0
20
40
60
80
Office hours
Out-of-office hours

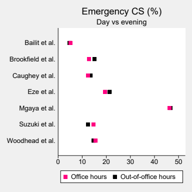
Emergency CS (%)
Day vs evening
Bailit et al.
Brookfield et al.
Caughey et al.
Eze et al.
Mgaya et al.
Suzuki et al.
Woodhead et al.
0
10
20
30
40
50
Office hours
Out-of-office hours

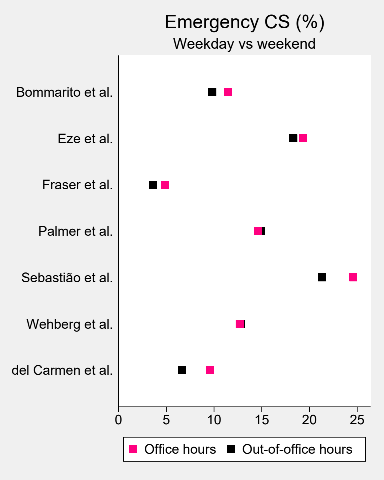
Emergency CS (%)
Weekday vs weekend
Bommarito et al.
Eze et al.
Fraser et al.
Palmer et al.
Sebastião et al.
Wehberg et al.
del Carmen et al.
0
5
10
15
20
25
Office hours
Out-of-office hours

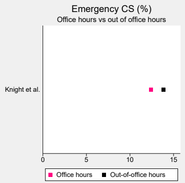
Emergency CS (%)
Office hours vs out of office hours
Knight et al.
0
5
10
15
Office hours
Out-of-office hours
